# Integrated transcriptomic and metabolomic analyses reveal the antibacterial mechanism of isocorydine against *Mycobacterium bovis*

**DOI:** 10.3389/fmicb.2025.1717499

**Published:** 2025-12-11

**Authors:** Xia Wang, Weichao Ma, Yijun Yuan, Xiang Gao, Tianyu Zhao, Peng Wang, Yanmei Liu, Tingpu Wang

**Affiliations:** 1 Gansu Provincial Universities Key Laboratory for Agricultural Microbiology, College of Bioengineering and Biotechnology, Tianshui Normal University, Tianshui, Gansu, China; 2 College of Chemical Engineering and Technology, Tianshui Normal University, Tianshui, Gansu, China

**Keywords:** *Mycobacterium bovis*, isocorydine, antibacterial mechanism, multi-omics, transcriptomics, metabolomics, efflux pumps, metabolic reprogramming

## Abstract

**Introduction:**

*Mycobacterium bovis*, the causative agent of zoonotic tuberculosis, poses a serious threat to public health and agriculture. This study investigates the antibacterial activity and mechanism of action of isocorydine (ICD), a natural alkaloid from *Dicranostigma leptopodum (Maxim.) Fedde* (*DLF*), against *M. bovis*.

**Methods:**

The minimum inhibitory concentration (MIC) of ICD was determined. Phenotypic changes were assessed through assays measuring cell wall/membrane integrity, ion leakage, extracellular pH, total lipid content, and electron microscopy. The global response of *M. bovis* to sub-inhibitory ICD was elucidated using transcriptomic and metabolomic profiling.

**Results:**

ICD exhibited potent antibacterial activity with a MIC of 400 μg/mL. It disrupted the cell wall and membrane, leading to ion leakage, pH alteration, reduced lipids, and severe ultrastructural damage. Transcriptomics revealed 66 differentially expressed genes, with significant upregulation of efflux pumps and TetR family regulators. Metabolomics identified 1,158 differential metabolites, indicating a profound metabolic rewiring characterized by depleted central carbon metabolites and accumulated fatty acids.

**Discussion/conclusion:**

Our results demonstrate that ICD exerts its antibacterial effect primarily by targeting the cell envelope, causing membrane disruption and energetic stress. *M. bovis* responds by activating efflux pumps and reprogramming its metabolism. This multi-omics study reveals the potential of ICD as an anti-mycobacterial agent and provides novel insights into the adaptive strategies of M. bovis under phytochemical stress.

## Introduction

Tuberculosis (TB), a disease of profound global health significance, continues to be a leading cause of mortality worldwide, with an estimated 1.3 million deaths annually (Harding, 2020). While *Mycobacterium tuberculosis* is the primary agent of human TB, its close relative, *Mycobacterium bovis*, presents a significant zoonotic threat. *M. bovis* is the causative agent of bovine tuberculosis (bTB), a disease that not only results in substantial economic losses to the livestock industry but also infects humans, typically through the consumption of unpasteurized dairy products or via aerosol transmission (Kanipe and Palmer, 2020; Kasir et al., 2023). The genomes of *M. tuberculosis* and *M. bovis* share 99.95% sequence identity (Garnier et al., 2003), yet a critical distinction lies in the innate resistance of *M. bovis* to pyrazinamide (PZA) (Esteban and Muñoz-Egea, 2016). This inherent resistance complicates standard anti-TB therapy, often necessitating prolonged treatment regimens and contributing to the challenges of TB control (Lange et al., 2022).

The growing crisis of antimicrobial resistance further complicates tuberculosis treatment. The standard first-line regimen for human TB, which is largely applicable to *M. bovis*, comprises isoniazid (INH), rifampicin (RIF), ethambutol (EMB), and pyrazinamide (PZA) (Nahid et al., 2016). These drugs act on a narrow set of essential bacterial processes: INH inhibits mycolic acid synthesis (Vilchèze and Jacobs, 2007), RIF binds RNA polymerase (Campbell et al., 2001), EMB impairs arabinogalactan polymerization (Telenti et al., 1997), and PZA, to which *M. bovis* is intrinsically resistant, disrupts membrane energy homeostasis (Zhang et al., 2013). The rise of multidrug-resistant (MDR) and extensively drug-resistant (XDR) strains of *M. tuberculosis* and *M. bovis* reveals a critical vulnerability in this reliance on a limited target set (World Health Organization, 2023). Widespread resistance mutations in katG (INH), rpoB (RIF), and embB (EMB) undermine drug efficacy and underscore the urgent need for new therapeutics with novel mechanisms to bypass existing resistance pathways (Zhang and Yew, 2015; Koul et al., 2011).

The current drug pipeline for TB remains insufficient, highlighting the importance of exploring alternative sources for lead compounds. In this context, natural products, particularly plant-derived phytochemicals, have historically been a rich reservoir of antimicrobial agents and offer a promising avenue for discovery (Kim et al., 2018; SeyedAlinaghi et al., 2025). Considering the critical role and structural complexity of the mycobacterial cell envelope, which serves as a primary action site for several first-line drugs while still containing underexplored vulnerabilities (Jankute et al., 2015), compounds targeting this essential structure through novel mechanisms present a particularly attractive strategy (Batt et al., 2020).

*Dicranostigma leptopodum (Maxim.) Fedde* (*DLF*), a plant used in traditional Chinese medicine for its anti-inflammatory properties, is a known source of diverse bioactive isoquinoline alkaloids (Wang et al., 2022; Yan et al., 2009). Among these alkaloids, isocorydine (ICD), an aporphine-type compound, has attracted attention for its documented pharmacological activities. Previous studies have primarily focused on its anti-cancer properties, demonstrating its efficacy in inducing apoptosis and cell cycle arrest in various cancer cell lines (Zhang et al., 2020; Sun et al., 2012). More recently, its anti-sepsis activity has been linked to the modulation of inflammatory pathways (Luo et al., 2022).

While alkaloid extracts from *DLF* have demonstrated broad-spectrum antibacterial activity against pathogens like *Staphylococcus aureus* and *Escherichia coli* (Zhao et al., 2008), the specific antibacterial potential of its principal alkaloid, ICD, against mycobacterial species remains entirely unexplored.

Preliminary phenotypic studies from our group have indicated that ICD exhibits potent bacteriostatic and bactericidal effects against *M. bovis*, causing significant damage to the cell envelope. However, the precise molecular targets of ICD and the mechanisms underlying its bactericidal outcome remain unknown. In the era of systems biology, omics technologies provide powerful tools to unravel complex host-pathogen and drug-pathogen interactions (Ahamad et al., 2022). Transcriptomics and metabolomics, in particular, can offer an unbiased, comprehensive view of the bacterial adaptive response, revealing key pathways involved in antibacterial activity and its mechanism of action (LeGouëllec et al., 2018; Mashabela et al., 2019).

Therefore, to bridge this knowledge gap, we undertook an integrated multi-omics approach to elucidate the antibacterial mechanism of ICD against *M. bovis*. This study aims to confirm the antibacterial activity of ICD through phenotypic assays, characterize the ultrastructural damage inflicted on *M. bovis* cells, and, most critically, define the global transcriptional and metabolic alterations that underpin its bactericidal activity. We hypothesize that ICD kills mycobacteria by disrupting the cell envelope, which provokes metabolic dysregulation that exceeds the bacterium’s compensatory capacity and leads to cell death. By integrating multi-omics datasets, this research aims to decipher this cascade of failure, providing novel insights into the mechanism of action of a promising phytochemical and revealing how its multi-target assault leads to a lethal outcome, ultimately contributing to the development of new therapeutic strategies against zoonotic tuberculosis.

## Materials and methods

### Separation and purification of isocorydine (ICD)

Total alkaloids were extracted from *DLF* and purified using acidic cationic resin, ICD was subsequently isolated from the total alkaloids through silica gel column chromatography. The mass of silica gel used was 80–100 times greater than the extracted alkaloids from *DLF*. The silica gel was activated with dichloromethane, and the resulting slurry was loaded into a chromatography column at a consistent rate. Once the dichloromethane reached the silica gel level, the *DLF* alkaloids dissolved in dichloromethane were added to the column until the liquid phase equalled the silica gel level. A layer of approximately 2 cm of sea sand was layered on top of the silica gel to facilitate the elution process. The elution of *DLF* alkaloids was achieved using a solvent mixture of dichloromethane, anhydrous methanol, and diethylamine in a volumetric ratio of 50:1:0.1. The fraction containing ICD was monitored using thin layer chromatography (TLC) with a developing solvent of dichloromethane and anhydrous methanol (20:1). Fraction collection ceased upon detecting a second product. The purified ICD solution was then concentrated and dried using rotary evaporation, producing solid ICD (Liu et al., 2011). High-performance liquid chromatography (HPLC) determined the ICD purity at 99.67% using an Agilent Technologies LC 1260 Infinity II series (Supplementary Figure S1). Chromatographic conditions included a Kromasil 100-5-C18 column (4.6 × 250 mm, 5 μm) with mobile phases of acetonitrile (A) and 0.2% acetic acid in water, pH 5.1 (B). The column was maintained at 35 °C, with a flow rate of 1.0 mL/min and detection at *λ* = 268 nm. The injection volume was 20.0 μL, and column pressure was 199 bar. The gradient elution profile was: A, 15–25% over 20 min, then increased to 80% over the subsequent 10 min.

### Bacterial strains and culture condition

The bacterial strain *M. bovis* CVCC68002 was obtained from the China Institute of Veterinary Drugs Control (Beijing, China). It was cultured at 37 °C in 7H9 liquid medium containing 0.47% 7H9, 0.05% glycerin and 0.005% Tween-80, sterilized at 121 °C for 15 min. After cooling to 55–60 °C, the medium was supplemented with 10% oleic acid-albumin-dextrose-catalase (OADC; Gene Optimal, Guangdong, China). Similarly, 7H10 medium containing 1.9% 7H10 and 0.05% glycerin, was enriched with 10% OADC. Stock solutions of ICD were prepared as follows: Solid ICD from *DLF* was dissolved in dimethyl sulfoxide (DMSO) to yield a 50 mg/mL primary stock. The stock was briefly ultrasonicated to ensure complete dissolution, sterile-filtered through a 0.22 μm membrane, aliquoted, and stored at −20 °C. For antibacterial assays, thawed aliquots were diluted in sterile water to produce working solutions, which were added to culture medium to attain the final concentrations indicated. The “0 μg/mL” ICD condition served as the vehicle control and contained 0.9% (v/v) DMSO; validation experiments confirmed that this concentration had no detectable effect on bacterial growth. *M. bovis* was incubated in either 7H9 or 7H10 medium at 37 °C, supplemented with 32 μg/mL rifampicin (positive control) and ICD at concentrations of 0, 150, 200, 250, 300, 350, 400 and 450 μg/mL.

### Assessment of antibacterial activity and growth kinetics

The antibacterial activity of ICD against *M. bovis* CVCC68002 was evaluated using a flask-based growth inhibition assay. All phenotypic assays reported here were performed with three independent biological replicates (*n* = 3) on separate occasions. A mid-exponential starter culture (OD₆₀₀ ≈ 0.6–0.8) was diluted into fresh 7H9 medium in sterile 100 mL Erlenmeyer flasks to achieve an initial OD_600_ of 0.01 in a final volume of 50 mL. To prevent bacterial aggregation and ensure homogeneous sampling, each flask contained five sterile glass beads (4 mm diameter). The diluted suspension was immediately treated with ICD (150–450 μg/mL), rifampicin (32 μg/mL, positive control), or an equivalent volume of DMSO (vehicle control); this time point was designated *T* = 0. Flasks were incubated at 37 °C with constant shaking at 180 rpm.

To monitor growth kinetics, 200 μL aliquots were aseptically withdrawn from each flask at predetermined intervals. Before each sampling, the entire 50 mL culture was homogenized by vigorously aspirating and expelling it ten times with a 25 mL sterile serological pipette to disperse any bacterial clumps. The optical density at 600 nm (OD₆₀₀) of the homogenized aliquots was then immediately measured using a microplate reader (Infinite M Plex, Tecan Trading AG). The minimal inhibitory concentration (MIC) was defined as the lowest concentration of ICD that inhibited ≥90% of bacterial growth after 7 days of incubation, compared to the vehicle control at the same time point. For the solid medium growth assay, cells were harvested from the liquid cultures, serially diluted in sterile saline, and spotted onto 7H10 agar plates, with or without ICD, to determine the number of colony-forming units (CFUs).

### Delayed-addition assay to determine optimal antibacterial timing

To assess how treatment timing affects ICD efficacy, a delayed-addition assay was conducted with three independent biological replicates (*n* = 3). A primary culture of *M. bovis* was prepared and diluted to an OD₆₀₀ of 0.01 in a large volume of fresh 7H9 medium, then incubated at 37 °C as the central reservoir. Instead of adding the drug at *T* = 0, ICD was introduced into separate sub-cultures at various time points post-inoculation: specifically, at 0, 1, 2, 3, and 4 weeks. At these intervals, aliquots from the main culture were transferred to new flasks containing pre-dissolved ICD at final concentrations of 0 (vehicle control), 200 (1/2 MIC), 400 (MIC), and 600 μg/mL (3/2 MIC). After each drug addition, the sub-cultures were incubated under the same conditions. Weekly samples from all sub-cultures were collected to measure optical density at 600 nm, following the previously described homogenization protocol, allowing us to monitor growth outcomes relative to ICD introduction timing.

### Release of cellular constituents

The release of cellular constituents into the supernatants was measured according to a modified protocol based on Paul et al. (2011). *M. bovis* cultures aged 3 weeks and grown in 50 mL of 7H9 medium, were collected by centrifugation at 6,000 g for 20 min. The cells were washed three times with sterile phosphate-buffered saline (PBS, pH 7.0) and resuspended in 50 mL PBS. These suspensions were treated with varying concentrations of ICD (1/2 MIC, MIC and 3/2 MIC) over intervals of 0, 12, 24, 36, 48 and 60 h. Samples of 2 mL were collected and centrifuged at 12,000 g for 10 min to eliminate cellular debris. The concentration of released constituents was quantified by measuring the absorbance at 260 nm of 1 mL supernatant using a UV-2450 UV/Vis Spectrophotometer (SHIMADZU International Trade, Shanghai). This assay was performed with three independent biological replicates (*n* = 3).

### Measurement of extracellular conductivity and extracellular pH

Extracellular conductivity was measured according to the method established by Lee et al. (1998), with a DDSJ-319L conductivity meter (Shanghai Thunder Magnetic Instrument Co., Ltd., Shanghai, China). The extracellular pH of *M. bovis* was determined using a FE28-Standard pH meter (Mettler-Toledo Instruments (Shanghai) Co., Ltd., Shanghai, China). *M. bovis* was inoculated into 50 mL of 7H9 liquid medium at a 1–2% inoculation rate and incubated on a shaker at 37 °C with a rotation speed of 200 rpm for 2 weeks. Post-incubation, the culture was centrifuged to collect bacterial cells, which were washed twice with 0.1 mol/L PBS (pH 7.2) and transferred to 20 mL of 50 mmol/L KCl solution for starvation at 4 °C for 12 h. Then, ICD at concentrations of 1/2 MIC, MIC and 3/2 MIC was added to the KCl solution at room temperature for 4 h. The cells were washed twice with distilled water and resuspended in 20 mL of 10% (w/v) glucose solution. Extracellular pH was measured at intervals of 0, 12, 24, 36, 48, and 60 h using a pH meter. Results were expressed as extracellular conductivity (mS/cm) and pH values at each time point. These measurements were conducted with three independent biological replicates (*n* = 3).

### Determination of lipid content

The total lipid content of *M. bovis* cells treated with ICD from *DLF* at concentrations of 1/2 MIC, MIC and 3/2 MIC was determined using the phosphovanillin method (Ahmad et al., 2011). Three-week-old cells grown in 50 mL 7H9 medium were harvested and centrifuged at 6000 g for 10 min. The samples were then freeze-dried for 4 h. Approximately 0.1 g of dried cells were homogenized with liquid nitrogen and extracted with 4.0 mL of methanol-chloroform-water mixture (2:1:0.8, v/v/v) in a clean test tube, followed by 30 min of vigorous shaking. The tubes were centrifuged at 6,000 g for 10 min to separate the phases. The lower phase was mixed with 0.2 mL saline solution and centrifuged again at 4,000 g for 10 min. A 0.2 mL aliquot of chloroform-lipid mixture was transferred to a new tube, and 0.5 mL H_2_SO_4_ was added. This mixture was heated in a boiling water bath for 10 min. Subsequently, 3 mL of phosphovanillin was added, and the solution was shaken vigorously and incubated at room temperature for 10 min. Absorbance was measured at 520 nm, and total lipid content was calculated based on a standard calibration curve established using cholesterol as the reference standard. The total lipid content was determined from three independent biological replicates (*n* = 3).

### Scanning electron microscopy (SEM)

Samples for SEM analysis were prepared from at least two independent biological replicates per treatment condition. Bacterial cultures in the logarithmic growth phase were cultivated in 7H9 medium and exposed to ICD from *DLF* at concentrations of 1/2 MIC, MIC and 3/2 MIC for 60 h. The cells were washed with PBS and fixed in 2.5% glutaraldehyde (pH 7.4) for 2 h. Following fixation, samples were washed three times with 0.1 M phosphate buffer (pH 7.2) and fixed in 1% osmic acid at 4 °C for 2 h. The cells were then subjected to a gradient dehydration process using a series of ethanol concentrations (30, 50, 70, 80, 95, and 100%, v/v) before undergoing critical point drying. The samples were sputter-coated with gold for 30 s and analyzed using a Gemini SEM 300 scanning electron microscope (Zeiss, Germany). For each replicate, multiple fields of view were examined to ensure the observed morphological features were representative. Control samples were bacterial cultures not treated with ICD.

### Transmission electron microscopy (TEM)

The preparation of samples for TEM analysis followed the same replication principle, being derived from at least two independent biological cultures per condition. The logarithmic growth phase bacteria cultures in 7H9 medium were treated with ICD from *DLF* at different concentrations (1/2 MIC, MIC and 3/2 MIC) for 60 h. The precipitated cells were fixed in 2.5% glutaraldehyde (pH 7.4) for 2 h and embedded in low melting point agarose. After fixation, the samples were washed three times with 0.1 M phosphate buffer (pH 7.2) and fixed in 1% osmic acid at 4 °C for 2 h. Cells underwent gradient dehydration using a series of ethanol concentrations of 30, 50, 70, 80, 95, and 100% (v/v). Subsequently, the cells were embedded in Epon-Araldite resin for penetration and polymerization. Then ultrathin sections were collected for microstructural analysis and counterstained with 3% uranyl acetate and 2.7% lead citrate. Multiple grid squares and fields of view were thoroughly screened for each independent sample to confirm the consistency of the ultrastructural observations. Observations were performed using a HT7800 transmission electron microscope (HITACHI, Japan).

### RNA isolation

Cells were cultured to late exponential phase, and 50 mL of each culture was harvested by centrifugation at 6,000 g for 5 min at 4 °C. The pellet was washed with double-distilled water (ddH_2_O) without RNase and stored at −80 °C for RNA extraction. Samples were mixed with 1 mL chloroform: methanol (3:1, v/v) for approximately 60 s. Subsequently, 5 mL Trizol (Invitrogen, Carlsbad, USA) was added, and the mixture was shaken vigorously for 15 s, then incubated at room temperature for 10 min. After centrifugation at 12,000 g for 15 min at 4 °C, the upper phase was transferred to a new tube and mixed with an equal volume of isopropanol. The sample was incubated overnight at −20 °C. Post-incubation, the samples were centrifuged at 12,000 g for 15 min at 4 °C, and the supernatant was discarded. The pellet was washed twice with 600 μL of 70% ethanol, each time centrifuged at 10,000 g for 5 min at 4 °C, with supernatant removal after each wash. Residual ethanol was removed by pipetting, and the pellet was air-dried, resuspended in 50 μL of RNase-free water, and stored at −80 °C. Total RNA for transcriptomic sequencing and qRT-PCR validation was extracted from three independent biological replicates per condition.

### Transcriptome analysis

*Mycobacterium bovis* cells from three independent biological replicates per group were precultured in 7H9 medium supplemented with 10% (wt/vol) OADC until reaching early exponential phase (OD600 ≈ 0.3). They were then transferred to fresh 7H9 medium, either without ICD from *DLF* or with 1/2 MIC of ICD, and incubated for 12 h. Total RNA was extracted, treated with a Turbo DNA-free kit (Ambion, Carlsbad, CA, USA) to remove genomic DNA contamination, and quantified using a Nanodrop spectrophotometer (Quawell, San Jose, CA, USA). RNA sequencing (RNA-Seq) was performed by the staff at Shanghai Gene-optimal Sci-Tech (Shanghai, China). Ribosomal RNA was removed using the Epicentre Ribo-Zero rRNA Removal Kit. First-strand cDNA was synthesized using random primers and ProtoScript II reverse transcriptase, and the second strand was synthesized with a second-strand synthesis enzyme mix. The resulting double-stranded cDNA fragments were purified using AMPure XP beads (Agencourt, Brea, CA, USA), processed with an end repair reaction, and ligated to sequencing adapters. After purification by agarose gel electrophoresis, suitable fragments were enriched by PCR amplification using a universal PCR primer and an index (X) primer. The qualified cDNA library was quantified using Qubit (Thermo Fisher Scientific, MA, USA) and a high-sensitivity DNA chip assay. The library sequencing was performed on an Illumina cBot Cluster Generation System using a TruSeq PE Cluster kit, and the samples were sequenced on an Illumina NovaSeq 6000 platform.

### Bioinformatic analysis of RNA-seq data

The comprehensive bioinformatic analysis was conducted using R software (version 3.6.3). Raw sequencing reads were aligned to the *M. bovis* AF2122/97 reference genome (NCBI Assembly GCF_000018765.1) using Bowtie2 (version 2.2.3). Gene-level read counts were generated using HTSeq (version 0.6.1) in union mode. Differential expression analysis was performed using the DESeq2 package (version 1.10.1) (Anders and Huber, 2010). The DESeq2 pipeline applies the DESeq median-of-ratios method for data normalization and employs a negative binomial generalized linear model for hypothesis testing. *p*-values were adjusted using the Benjamini-Hochberg method to control the false discovery rate (FDR). A dual-threshold strategy was employed for differential gene identification. To capture the full spectrum of transcriptional perturbations, a broad set of differentially expressed genes (DEGs) was defined using a threshold of FDR < 0.05 and an absolute log₂ fold change (|log_2_FC|) > 0; this set was used for all Gene Ontology (GO) and Kyoto Encyclopedia of Genes and Genomes (KEGG) pathway enrichment analyses. Subsequently, to define a high-confidence core set of DEGs for experimental validation, a more stringent threshold of FDR < 0.05 and |log_2_FC| > 1 was applied. The dataset has been deposited in the NCBI Sequence Read Archive under the accession number PRJNA1320159 and is now publicly accessible. A complete cross-reference of all DEGs with their associated significant GO terms and KEGG pathways is provided in Supplementary Table S4.

### qRT-PCR analysis

To validate the RNA-seq output, twelve DEGs were selected and analyzed by qRT-PCR. This analysis was conducted using three independent biological replicates, with each replicate measured in technical quadruplicate. Total RNAs were extracted as previously described, and cDNA synthesis was conducted using a Prime-Script RT reagent kit (TaKaRa, Shiga, Japan) following the manufacturer’s protocol. This cDNA served as the template to assess the transcriptional status of target genes. For qRT-PCR, *M. bovis* cells were grown to early exponential phase (OD600 ≈ 0.3) in 7H9 medium. Cell pellets from 150 mL cultures were transferred to equal volumes of 7H9 medium with no ICD from *DLF*, or with 1/2MIC or MIC of ICD for 12 h. Cells were then collected, washed, and stored at −80 °C for RNA extraction. Quantitative PCR was conducted on a LightCycler 96 Real-Time PCR System (Roche, Basel, Switzerland) using SYBR green supermix (TaKaRa, Shiga, Japan). Data analysis employed the ΔΔCT method, normalized to the Sigma A (*M.b_GM002863*) rRNA gene as an endogenous control. Each experiment included triplicate biological and technical repeats. Primer sequences for qRT-PCR are listed in Supplementary Table S2.

### Untargeted metabolomics analysis

#### Sample preparation and LC–MS analysis

Cells were collected after 12 h of cultivation under different ICD from *DLF* concentrations (0 g/L, 1/2MIC). Metabolite extraction and LC–MS analysis were performed by Shanghai Gene-optimal Sci-Tech (Shanghai, China). Each sample received 1 mL of a methanol: acetonitrile: water solution (2:2:1, v/v), transferred to an EP tube, vortexed, and subjected to mechanical disruption in an ice bath for 20 min. The mixture was then filtered through a 0.22 μm organic membrane. Samples were incubated at −20 °C for 1 h to precipitate proteins, centrifuged at 13,000 rpm at 4 °C for 15 min, and filtered again. The supernatant was collected and dried for LC–MS analysis. Metabolic profiling was conducted using an Agilent 129 Infinity LC system (Agilent Technologies, Santa Clara, CA, USA) and an AB Triple TOF 560/660 (SCIEX, Framingham, MA, USA). Profiling was performed using six independent biological replicates per condition (0 g/L and 1/2 MIC ICD).

#### Data processing and statistical analysis

##### Data preprocessing and metabolite identification

Raw mass spectrometry data were converted to mzXML format using ProteoWizard software. The data were then processed with the XCMS package in R for tasks such as peak picking, alignment, retention time correction, and peak area extraction. Metabolite identification involved matching both accurate mass (with a mass tolerance of less than 25 ppm) and MS/MS spectra against databases built in the laboratory.

##### Multivariate statistical modeling and validation

Before analysis, the processed peak intensity data underwent Pareto scaling. Initially, an unsupervised Principal Component Analysis (PCA) was conducted to visualize inherent data clusters and identify potential outliers. Following this, a supervised Orthogonal Partial Least Squares-Discriminant Analysis (OPLS-DA) was carried out using the ropls package in R to enhance group separation. The model’s robustness and predictive capability were rigorously evaluated through a permutation test with 200 permutations to prevent overfitting.

##### Criteria for identifying differential metabolites

Differential metabolites were identified using a dual-criterion filter to ensure both statistical significance and their contribution to group separation. This involved a Variable Importance in Projection (VIP) score of ≥1.0 from the OPLS-DA model and a *p*-value of <0.05 from a two-tailed Student’s *t*-test.

##### Pathway enrichment analysis

We performed pathway enrichment analysis on the significantly altered metabolites using the MBROLE 2.0 web server, referencing the Kyoto Encyclopedia of Genes and Genomes (KEGG) database. A hypergeometric test was employed, and pathways with a *q*-value < 0.05, adjusted for false discovery rate (FDR), were deemed statistically significantly enriched.

### Statistical analysis

All comparisons between two independent groups were conducted with the nonparametric Mann–Whitney U test (Wilcoxon rank-sum test). This test was chosen because it does not assume normality and is robust for the sample sizes used in this study (three to six biological replicates per group across all experiments). Data are reported as mean ± standard deviation. Significance in figures is indicated as follows: *p < 0.05; **p < 0.01.

## Results

### Effect of isocorydine (ICD) on *Mycobacterium bovis* growth

**Figure 1:**
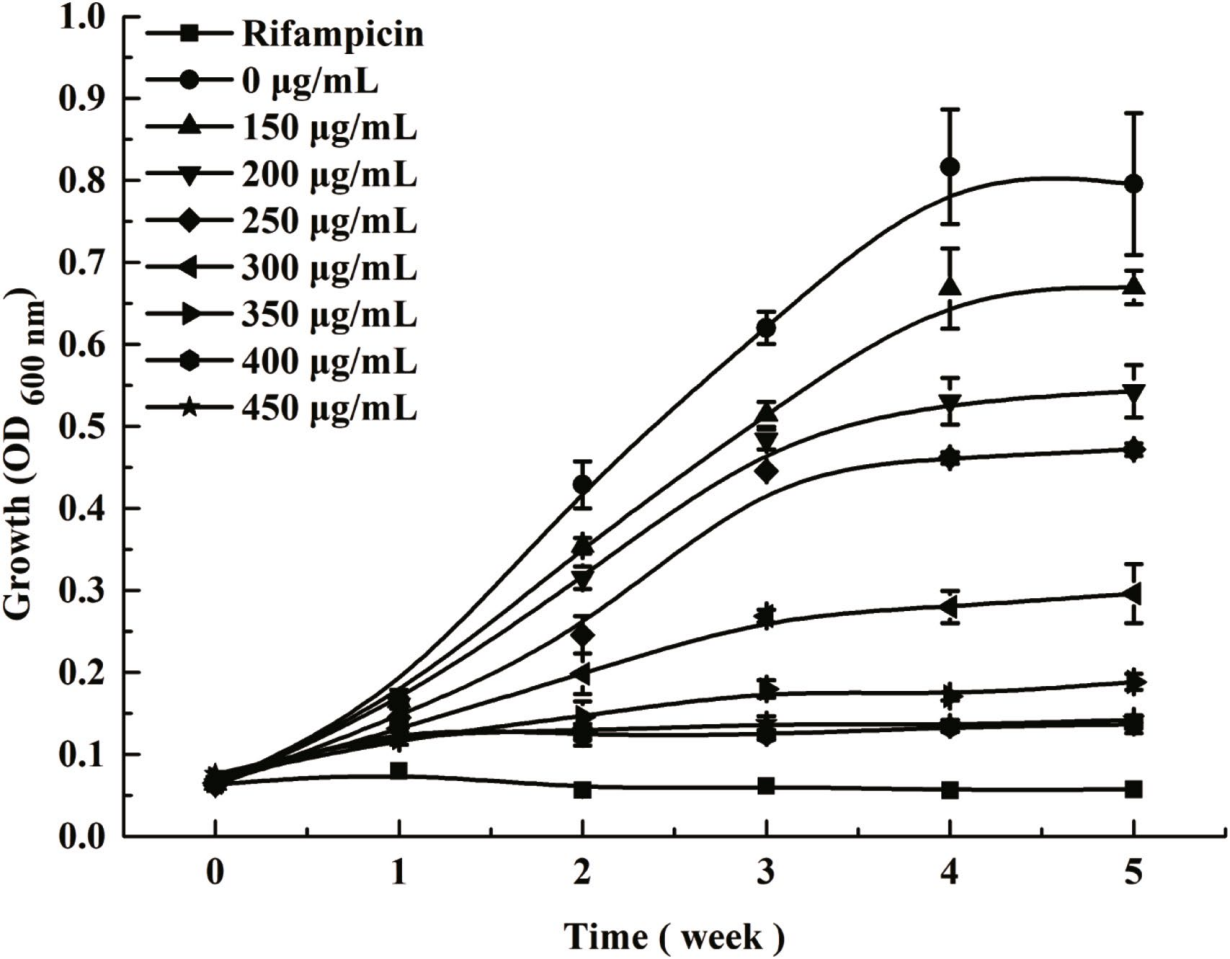
Growth analysis of *M. bovis* in response to isocorydine (ICD). Bacterial growth was monitored in 7H9 medium containing the indicated concentrations of ICD (0, 150, 200, 250, 300, 350, 400 and 450 μg/mL). The “0 μg/mL” data represents the vehicle control group. Data are presented as the mean ± SD (*n* = 3).

To investigate the inhibitory effects of ICD from *DLF* on the growth of *M. bovis*, different concentrations of ICD were added to the culture medium (Figure 1). The addition of 150 μg/mL ICD delayed the logarithmic growth phase and slightly reduced final biomass. Increasing the concentration to 300 μg/mL significantly inhibited the growth of *M. bovis*, further reducing biomass. Complete growth suppression occurred at 400 μg/mL, with no significant difference observed between 400 μg/mL and 450 μg/mL concentrations. This inhibitory effect of ICD was consistent in both liquid and solid culture medium (Supplementary Figure S2). These results demonstrate that higher ICD concentrations more effectively inhibit *M. bovis*, with a minimum inhibitory concentration (MIC) of 400 μg/mL. To further investigate the antibacterial effects of ICD from *DLF* against *M. bovis*, we evaluated its bacteriostatic rate and IC_50_ value. At 50 μg/mL, ICD showed no significant effect on the *M. bovis* growth. However, as the concentration increased, the bacteriostatic rate was calculated (Supplementary Table S1). At a concentration of 400 μg/mL, ICD achieved a 100% inhibition rate. The IC50 value was determined to be 198.04 μg/mL.

### Determination of the optimal antibacterial period of ICD on *M. bovis*

**Figure 2:**
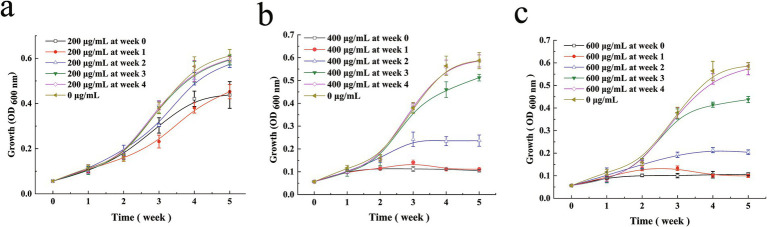
Determination of the optimal antibacterial exposure window for ICD against *M. bovis* using a delayed-addition assay. A central bacterial culture was maintained without treatment. At the indicated time points (0, 1, 2, 3, and 4 weeks), aliquots were taken to establish separate subcultures, which were then supplemented with ICD for the remainder of the experiment. For each growth curve, data points prior to the addition time reflect growth of the central untreated culture, whereas points after the addition show the effect of ICD. Growth curves are shown for ICD at **(a)** 200 (1/2 MIC), **(b)** 400 (MIC), and **(c)** 600 (3/2 MIC) μg/mL. Data are presented as mean ± SD (*n* = 3).

To further characterize the bacteriostatic properties of ICD from *DLF* on *M. bovis*, a delayed-addition assay was performed wherein ICD was added at different stages of the bacterial growth cycle (Figure 2). At a concentration of 1/2 MIC (200 μg/mL), ICD significantly reduced biomass by approximately 40% when added in the 0 and 1st week (Figure 2a). The addition of ICD in the 2nd week showed slight, non-significant growth inhibition. No effect was observed when ICD was added in the 3rd and 4th weeks. At the MIC concentration (400 μg/mL), ICD demonstrated a significant bacteriostatic effect when applied during the 0, 1st, and 2nd weeks, maintaining this effect for up to 5 weeks under both experimental conditions. The addition of ICD at MIC concentration during the 3rd and 4th weeks did not affect *M. bovis* growth (Figure 2b). Similarly, at a concentration of 3/2 MIC (600 μg/mL), ICD inhibited *M. bovis* growth when added in the 1st and 2nd weeks, but showed no effect in the 3rd or 4th weeks (Figure 2c). Thus, *M. bovis* is most sensitive to ICD during the early to mid-logarithmic growth phases.

### Release of cell constituents

**Figure 3:**
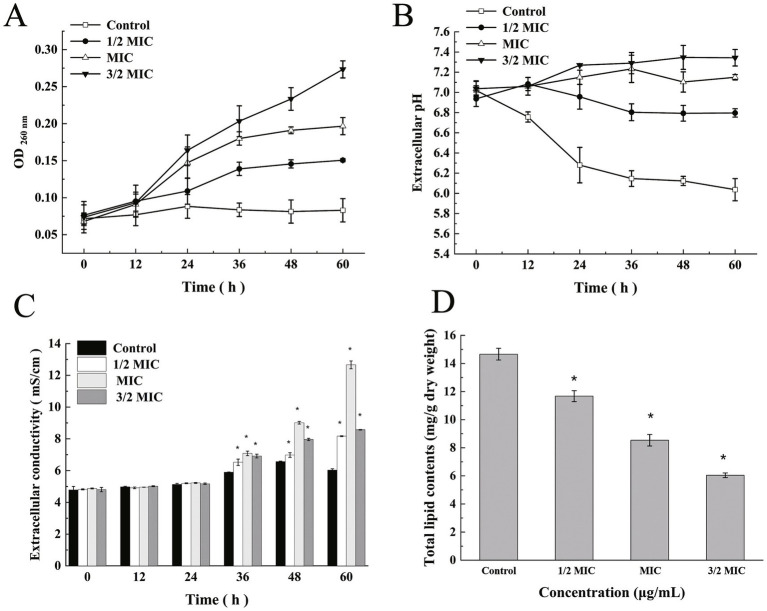
Examination of physiological changes in *M. bovis* induced by ICD. **(A)** Release of 260 nm-absorbing material, indicative of cell lysis. **(B)** Alterations in extracellular pH. **(C)** Changes in extracellular conductivity. **(D)** Total lipid content. Data are presented as the mean ± SD from three independent biological replicates (*n* = 3). Statistical significance was determined using the Mann–Whitney U test (*p < 0.05, **p < 0.01) compared to the untreated control at the corresponding time point.

We measured the release of cell constituents from *M. bovis* treated with ICD over 60 h. A significant increase (*p* < 0.05) in the release of 260 nm-absorbing materials was observed immediately upon treatment (Figure 3A). Over time, the release of 260 nm absorbing material significantly increased (*p* < 0.05). After 60 h, the OD260 value for *M. bovis* treated with 1/2 MIC of ICD was 0.295, significantly higher (*p* < 0.05) than the control (0.083), but lower (*p* < 0.05) than those treated with MIC (0.197) and 3/2 MIC (0.273). Overall, OD260 values showed a moderate upward trend with increasing ICD concentration.

### Extracellular pH

The extracellular pH of *M. bovis* cells exposed to ICD from *DLF* is presented in Figure 3B. In the control group, a sharp decrease in extracellular pH was observed, with a gradual decline between 36 to 60 h. After incubation with ICD at 1/2 MIC for 60 h, the extracellular pH of *M. bovis* suspensions was 6.80, significantly higher than that of the control group (6.03) (*p* < 0.05). Although the overall pH decreased, the reduction was less pronounced than in the control. After 60 h with ICD at MIC and 3/2 MIC, the extracellular pH values were 7.15 and 7.34, respectively, both significantly higher than that of the 1/2MIC (6.80) (*p* < 0.05).

### Extracellular conductivity

The results of extracellular conductivity from *M. bovis* cells treated with ICD from *DLF* at concentrations of 1/2 MIC, MIC and 3/2 MIC over 0–60 h are shown in Figure 3C. After 30 min of exposure, extracellular conductivity values were 0.253, 0.308, and 0.292 μS/cm, respectively, significantly higher than the control (0.19 μS/cm) (*p* < 0.05). This effect intensified with prolonged exposure. At 60 h, conductivity increased to 0.417, 0.457 and 0.866 μS/cm for 1/2 MIC, MIC and 3/2 MIC, respectively.

### Total lipid content

The effect of ICD from *DLF* on the total lipid content of *M. bovis* cells is shown in Figure 3D. A significant reduction (*p* < 0.05) was observed with increasing concentrations of ICD in total lipid content of *M. bovis* cells. After 48 h of incubation at 1/2 MIC, MIC and 3/2 MIC concentrations, the lipid contents were 11.67, 8.53 and 6.04 mg/g dry weight, respectively, all significantly lower than the control value of 14.66 mg/g dry weight.

### Scanning electron microscopy (SEM)

**Figure 4:**
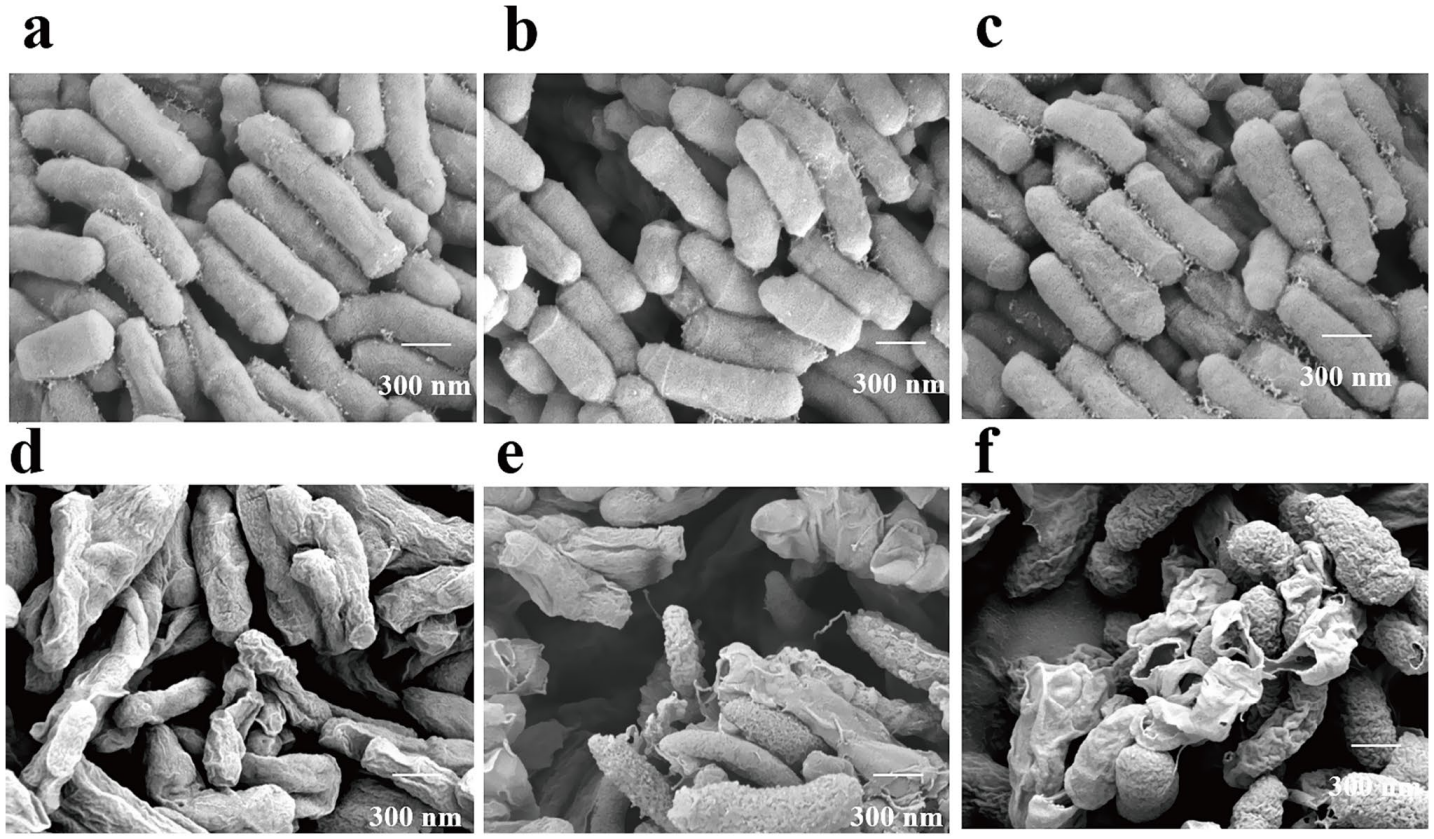
Scanning electron micrographs of *M. bovis* after 60 h of ICD treatment. **(a–c)** Untreated control cells. **(d–f)** Cells treated with ICD at 1/2 MIC, MIC, and 3/2 MIC, respectively. Samples are representative of at least two independent biological replicates per condition.

The effects of the ICD from *DLF* on the morphology of *M. bovis* were examined using SEM (Figure 4). Control samples grown on 7H9 medium displayed typical rod-shaped bacterial morphology, with homogeneous hyphae and intact structures (Figures 4a–c). After 60 h with 1/2 MIC of ICD, bacterial cells exhibited shrinkage, invagination, and distortion, without obvious cell rupture. In contrast, treatment with MIC and 3/2 MIC of ICD for 60 h resulted in dehydrated, distorted cells with ruptured membranes and walls, irregular structures, and inhomogeneous appearances, with protoplasts visibly escaping (Figures 4d–f). The severity of these changes increased with higher concentrations of ICD, indicating significant destruction of *M. bovis* cell membranes and walls.

### Transmission electron microscopy (TEM)

**Figure 5:**
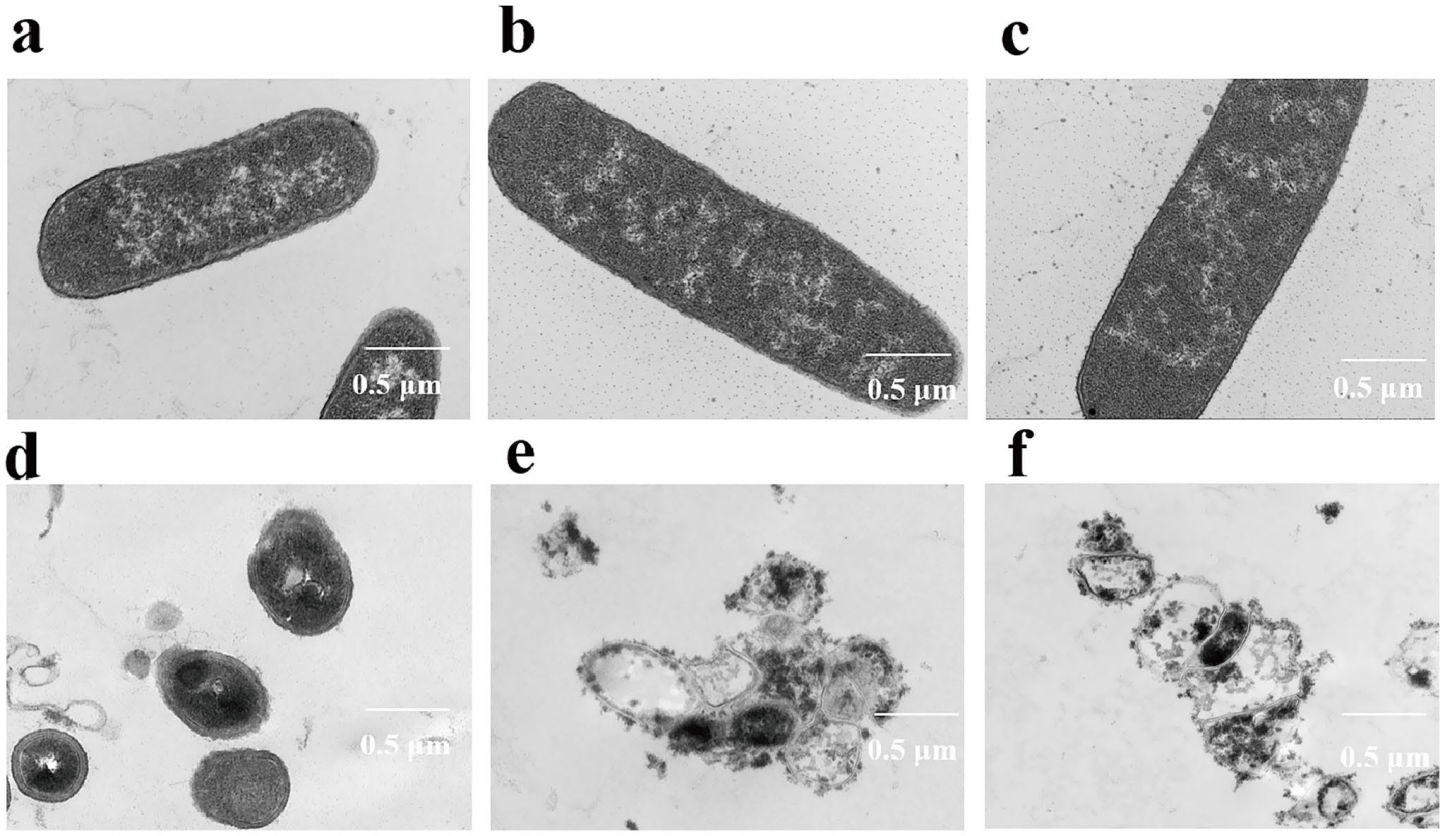
Transmission electron micrographs of *M. bovis* after 60 h of ICD treatment. **(a–c)** Untreated control cells. **(d–f)** Cells treated with ICD at 1/2 MIC, MIC, and 3/2 MIC, respectively. Samples are representative of at least two independent biological replicates per condition.

The internal structures of *M. bovis* treated with ICD from *DLF* at concentrations of 1/2 MIC, MIC and 3/2 MIC for 60 h were observed using TEM (Figure 5). Control samples showed normal morphology, characterized by smooth cell walls and membranes, with a homogeneous cytoplasm (Figures 5a–c). However, Cells exposed to 1/2MIC ICD displayed indistinct cell boundaries and uneven cell wall and membranes, though internal changes were minimal (Figure 5d). Treatment with MIC and 3/2 MIC ICD caused significant ultrastructure damage including shriveled, ruptured or absent plasmalemma, and dissolution of intracellular substances (Figures 5e,f). These effects intensified with higher ICD concentrations, indicating that ICD from *DLF* severely damages *M. bovis* cell membranes and walls, leading to cytoplasmic content release.

### Effects of ICD stress on gene expression in *M. bovis*

**Figure 6:**
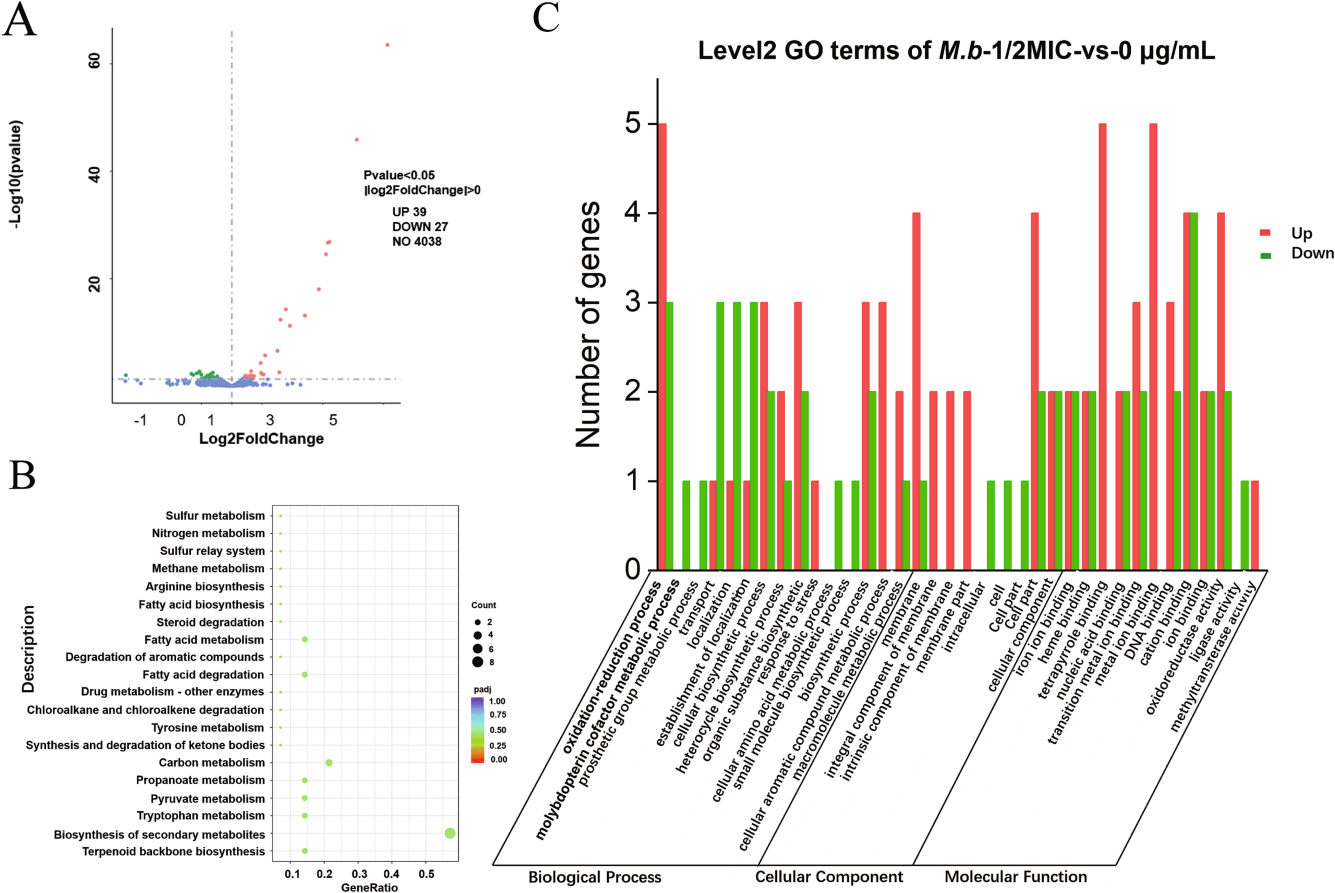
Transcriptomic profiling of *M. bovis* under ICD stress. **(A)** Volcano plot displaying all 66 differentially expressed genes (DEGs) identified using a threshold of FDR < 0.05 and |log₂FC| > 0. Up-regulated and down-regulated genes are indicated in red and green, respectively. **(B)** Kyoto Encyclopedia of Genes and Genomes (KEGG) pathway and **(C)** Gene Ontology (GO) enrichment analyses performed on this broad set of 66 DEGs. All analyses were based on three independent biological replicates per group.

**Figure 7:**
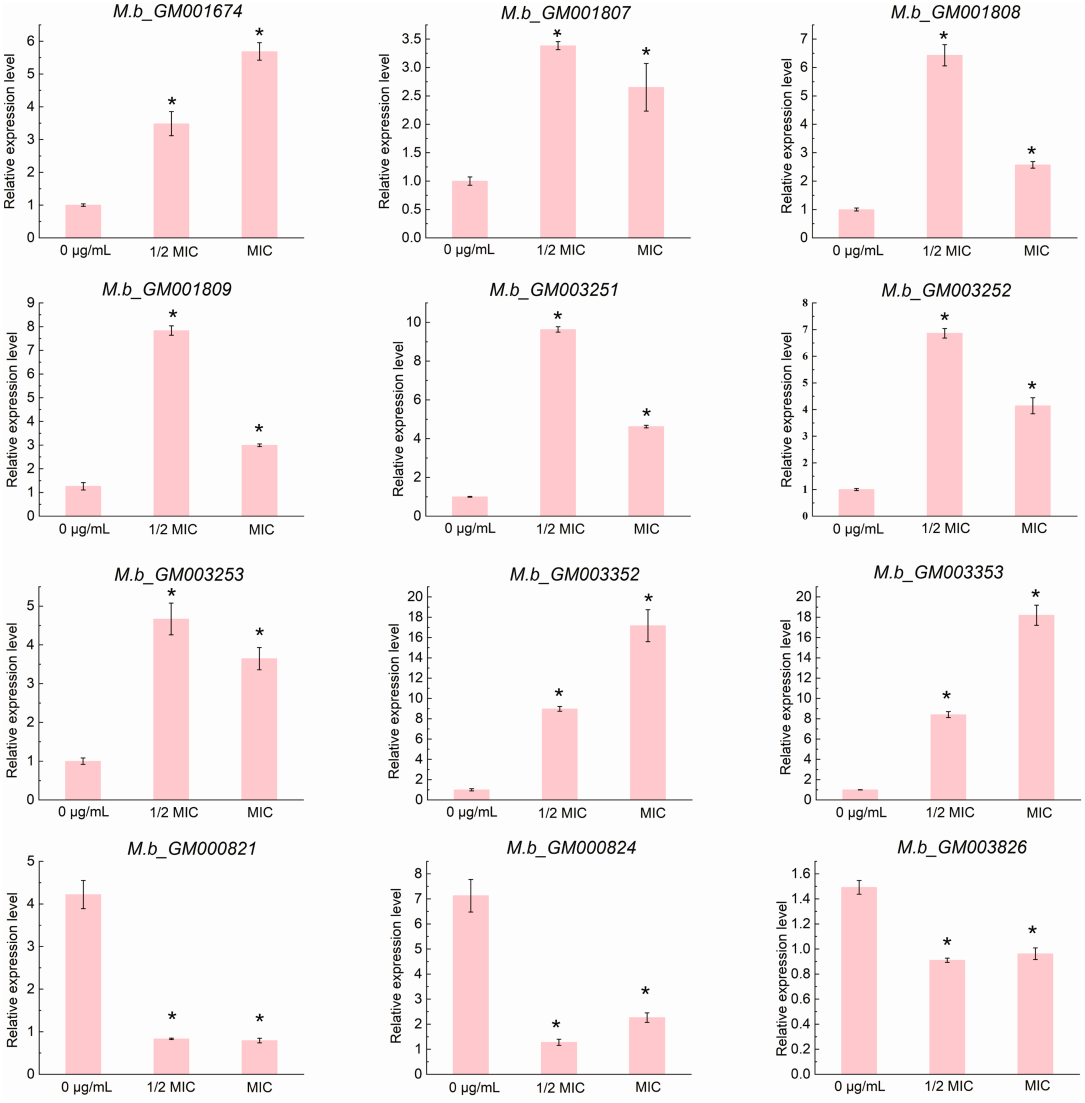
qRT-PCR validation of the expression profiles of 12 structural genes. Data are presented as the mean ± SD from three biological replicates, with each replicate measured in quadruplicate (*n* = 3). The asterisk (*) indicates a statistically significant difference with a p-value of less than 0.05, as determined by the Mann-Whitney U test (Wilcoxon rank-sum test).

Transcriptomic analysis was conducted to elucidate the tolerance mechanism of *M. bovis* under ICD-induced stress. Gene expression profiles were compared between cultures exposed to 1/2 MIC of ICD and untreated controls. Differential gene expression analysis was performed using DESeq2. We employed a dual-threshold strategy to map the transcriptional landscape comprehensively. Firstly, we initially identified a broad set of differentially expressed genes (DEGs) using a threshold of FDR < 0.05 and an absolute log₂ fold change (|log_2_FC|) > 0. This analysis revealed 66 DEGs, with 39 up-regulated and 27 down-regulated, as shown in the volcano plot (Figure 6A). This set of 66 DEGs was utilized for all subsequent Gene Ontology (GO) and Kyoto Encyclopedia of Genes and Genomes (KEGG) pathway enrichment analyses (Figures 6B,C; Supplementary Table S3). To define a high-confidence core set of genes with significant expression changes for functional interpretation and experimental validation, we applied a more stringent threshold (FDR < 0.05 and |log_2_FC| > 1). This refined the core set to 22 DEGs, comprising 15 up-regulated and 7 down-regulated genes, as detailed in Table 1. The expression of genes within this core set, associated with stress response and detoxification pathways, including those encoding TetR family transcriptional regulators, ABC transporters, and MMPL family proteins, was significantly upregulated in the 1/2 MIC ICD treatment group. These included *M.b_GM003352* and *M.b_GM001807* (TetR family regulators), *M.b_GM001808* (ABC-2 type transporter), *M.b_GM001809* (ABC transporter), *M.b_GM003253* (TetR family), *M.b_GM003252* (small multidrug resistance protein), *M.b_GM003251* (DoxX), and *M.b_GM001674* (MMPL family). Conversely, genes such as *M.b_GM003826* (excreted virulence factor EspC, type VII ESX diderm), *M.b_GM000821* (NTF2 domain protein), and *M.b_GM000824* (protein of unknown function) were significantly down-regulated. To validate the RNA-seq results, qRT-PCR was performed on twelve selected DEGs from this high-confidence core set. For qRT-PCR validation, cells were treated with 0 g/L, 1/2 MIC, or MIC of ICD for 12 h. The expression trends of nine up-regulated genes (*M.b_GM001674*, *M.b_GM001807*, *M.b_GM001808*, *M.b_GM001809*, *M.b_GM003251*, *M.b_GM003252*, *M.b_GM003253*, *M.b_GM003352*, and *M.b_GM003353*) and three down-regulated genes (*M.b_GM003826*, *M.b_GM000821*, and *M.b_GM000824*) were consistent with the transcriptomic data (Figure 7).

**Table 1:** The significantly differentially expressed genes in the 1/2MIC ICD treatment group and 0 g/L ICD control group.

Up/Down regulated	Gene ID	ICD-Treated_-mean	Control_-mean	Log2(FC)	*p*-value	Symbol
Up	*M.b_GM003352*	2917.728	1451.909	1.007474	0.006062	PF00440: Bacterial regulatory proteins, tetR family
	*M.b_GM003353*	13068.68	6341.463	1.04335	0.006915	PF00848: Ring hydroxylating alpha subunit (catalytic domain) | PF00355: Rieske [2Fe-2S] domain
	*M.b_GM000598*	753.1648	352.3796	1.096849	1.98E-06	PF01209: ubiE/COQ5 methyltransferase family
	*M.b_GM000359*	1025.553	361.2512	1.505232	2.74E-07	PF07992: Pyridine nucleotide-disulfide oxidoreductase
	*M.b_GM001808*	281.385	94.51791	1.568028	0.002719	PF01061: ABC-2 type transporter
	*M.b_GM003254*	302.7897	99.18411	1.605775	4.25E-13	PF05305: Protein of unknown function (DUF732)
	*M.b_GM001674*	693.6713	201.6787	1.781382	5.17E-15	PF03176: MMPL family
	*M.b_GM001807*	220.3509	58.16656	1.919079	6.14E-12	PF00440: Bacterial regulatory proteins, tetR family
	*M.b_GM001809*	517.9266	97.01511	2.409215	7.81E-14	PF00005: ABC transporter
	*M.b_GM003251*	403.5499	55.57222	2.87042	9.42E-19	PF07681: DoxX
	*M.b_GM003253*	727.9755	84.08253	3.106218	2.98E-25	PF00440: Bacterial regulatory proteins, tetR family
	*M.b_GM000599*	4299.181	479.0364	3.167835	2.01E-27	PF05305: Protein of unknown function (DUF732)
	*M.b_GM001673*	256.2278	27.28371	3.219165	1.41E-27	PF05423: Mycobacterium membrane protein
	*M.b_GM003252*	869.1134	50.35078	4.12346	1.33E-46	PF00893: Small Multidrug Resistance protein
	*M.b_GM000600*	19,321	550.79	5.134085	2.98E-64	PF13847: Methyltransferase domain
Down	*M.b_GM003826*	447.9092	899.5288	−1.00494	0.007273	PF10824: Excreted virulence factor EspC, type VII ESX diderm
	*M.b_GM001283*	86.42393	180.7311	−1.06117	0.003125	PF00668: Condensation domain
	*M.b_GM000316*	119.9911	251.801	−1.06637	0.001867	Protein of unknown function
	*M.b_GM001516*	707.7793	1583.616	−1.16131	0.004322	Protein of unknown function
	*M.b_GM001008*	16.63331	40.1141	−1.27362	0.00836	PF00005: ABC transporter
	*M.b_GM000821*	24.18507	61.42629	−1.3337	0.005151	PF02136: Nuclear transport factor 2 (NTF2) domain
	*M.b_GM000824*	0.737089	8.428985	−3.49399	0.009532	Protein of unknown function

Gene Ontology (GO) enrichment analysis of the broad set of 66 DEGs categorized the DEGs into three functional domains: biological process, cellular component, and molecular function. In biological processes, DEGs were primarily associated with oxidation–reduction, cellular biosynthetic processes, and organic substance biosynthesis. Within cellular components, DEGs were predominantly localized to membranes and other cellular structures. For molecular function, the genes were mainly involved in ion binding, DNA binding, oxidoreductase activity, and nucleic acid binding (Figure 6C). The Kyoto Encyclopedia of Genes and Genomes (KEGG) pathway analysis of the broad set identified significant enrichment across various metabolic and biosynthetic pathways (Figure 6B; Supplementary Table S3). These pathways include the biosynthesis of secondary metabolites and the metabolism of tryptophan, terpenoids, pyruvate, propanoate, carbon, fatty acids, tyrosine, and amino acids. Additionally, the analysis highlighted degradation pathways for fatty acids, chloroalkanes, aromatic compounds, steroids, benzoate, valine, leucine, isoleucine, butanoate, lysine, and ketone bodies. Also enriched were glyoxylate and dicarboxylate metabolism, two-component systems, ABC transporters, glycolysis/gluconeogenesis, folate biosynthesis, purine and pyrimidine metabolism, sulfur and methane metabolism, and nitrogen assimilation pathways.

### Metabolomic analysis of intracellular metabolite alterations in *M. bovis* under ICD stress

**Figure 8:**
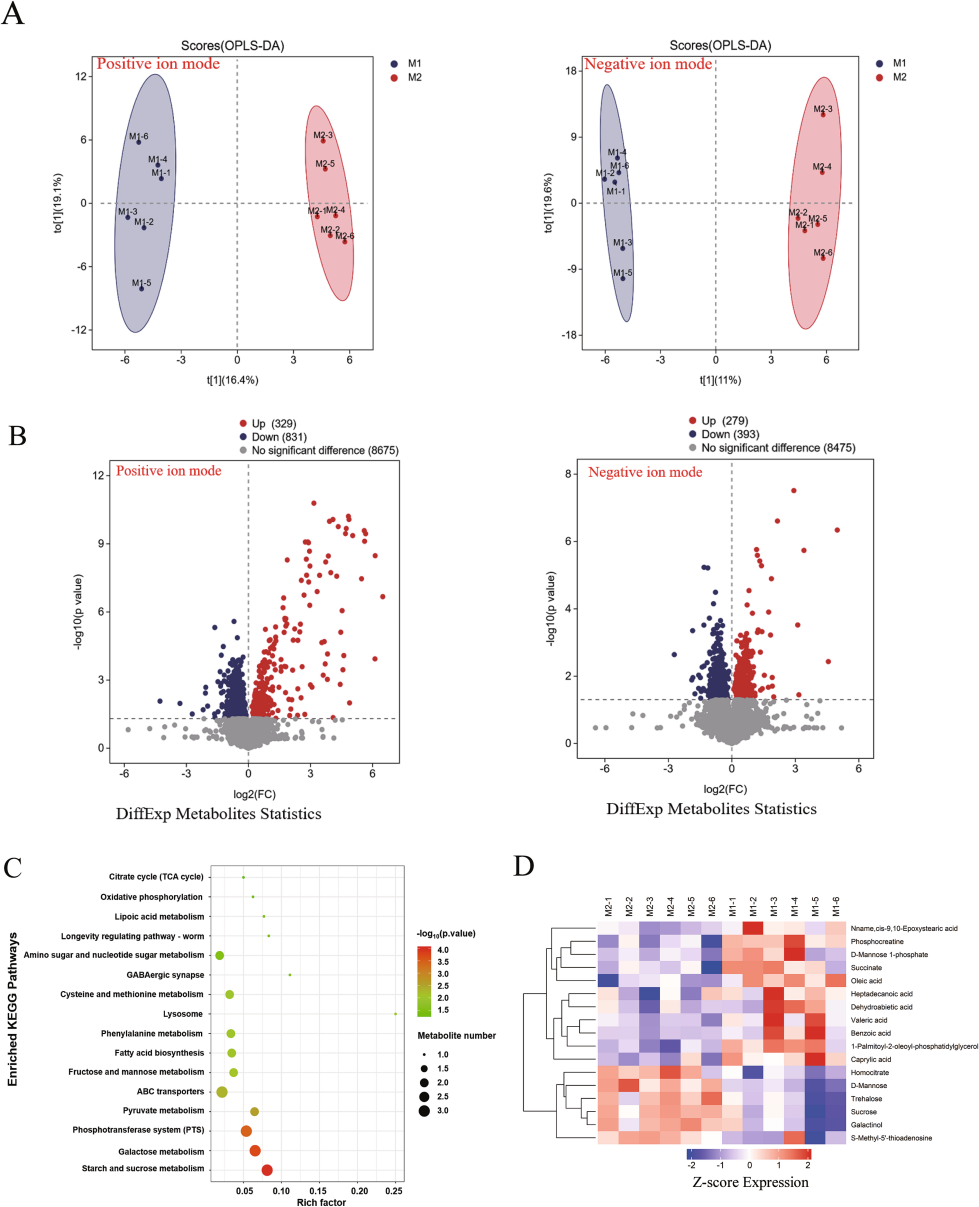
Metabolomic analysis of intracellular metabolite alterations in *M. bovis* under ICD stress. **(A)** OPLS-DA scores plot showing clear separation between the 1/2 MIC ICD-treated and control (0 μg/mL) groups in both positive and negative ion modes. **(B)** Count of significantly up-regulated and down-regulated differential metabolites. **(C)** KEGG pathway enrichment analysis of differential metabolites. The dot size represents the number of metabolites mapped to the pathway, and the color intensity corresponds to the significance level (−log10(q-value)). **(D)** Heatmap of hierarchical clustering of differential metabolites identified in negative ion mode. The color bar, ranging from blue to red, indicates the Z-score of relative metabolite abundance, highlighting metabolites with levels below (blue) or above (red) the mean expression across samples. All metabolomic profiling was performed using six independent biological replicates per group.

To explore the metabolic adaptations of *M. bovis* to ICD, we performed a comparative metabolomic analysis between untreated controls (0 g/L) and cells subjected to 1/2MIC of ICD stress. A comprehensive profiling of intracellular metabolites was conducted using LC–MS in both positive and negative ion modes. Initially, an unsupervised Principal Component Analysis (PCA) was conducted, followed by a supervised Orthogonal Partial Least Squares-Discriminant Analysis (OPLS-DA). The OPLS-DA model was employed, and metabolites were ranked by their Variable Importance in Projection (VIP) scores to identify those contributing most to group discrimination. OPLS-DA revealed a clear separation between the metabolic profiles of the ICD treated and control groups (Figure 8A), indicating a significant stress induced metabolic shift. In positive ion mode, 329 significantly up-regulated and 831 down-regulated differential metabolites (DMs, differentially expressed metabolites) were identified. Among these, S-Adenosyl-L-homocysteine and Isomaltose were confidently annotated (Table 2). In negative ion mode, 279 metabolites were up-regulated and 393 were down-regulated (Figure 8B), with 13 annotated DMs, including 1-Palmitoyl-2-oleoyl-phosphatidylglycerol, Phosphocreatine, Oleic acid, Sucrose, Caprylic acid, Homocitrate, Galactinol, Trehalose, Valeric acid, Succinate, Benzoic acid, and S-Methyl-5′-thioadenosine (Table 2).

**Table 2:** Subset of metabolites differentially expressed in the 1/2MIC ICD treatment group and 0 g/L ICD control group.

Mode	Protein ID	DMs name	Log2 (Fold change)	*p*-value	VIP	Adduct
POS	M385T386	S-Adenosyl-L-homocysteine	2.507685503	1.87504E-05	1.093741874	(M + H) +
	M360T397	Isomaltose	1.34066424	0.01642038	1.78348912	(M + H) +
NEG	M179T394	D-Mannose	1.329389517	0.000607792	1.157329051	(M-H)-
	M748T80	1-Palmitoyl-2-oleoyl-phosphatidylglycerol	0.588272782	0.001629239	9.144366426	(M-H)-
	M421T484	Phosphocreatine	0.664788655	0.003144902	4.204572125	(2 M-H)-
	M281T137	Oleic acid	0.717190142	0.00326465	3.173356603	(M-H)-
	M341T398	Sucrose	1.314062354	0.004095456	12.4652286	(M-H)-
	M143T53	Caprylic acid	0.552623959	0.006338657	15.25873772	(M + CH3COO)-
	M205T490	Homocitrate	1.448039403	0.00663391	1.450415775	(M + Na-2H)-
	M401T398	Galactinol	1.338342837	0.007529864	8.456286026	(M-H)-
	M363T398	Trehalose	1.248585164	0.008059616	1.287541739	(M-H)-
	M101T101	Valeric acid	0.669743319	0.027040027	3.460477928	(M-H)-
	M117T387_2	Succinate	0.815186699	0.028620787	1.999104355	(M-H)-
	M121T129	Benzoic acid	0.675867308	0.037777918	4.833226317	(M-H)-
	M296T98	S-Methyl-5′-thioadenosine	1.2053178	0.040977242	1.697318629	(M-H)-

DMs, differentially expressed metabolites; VIP, variable importance in the projection from the OPLS-DA model; Adduct, the ion adduct form detected by mass spectrometry (e.g., (M + H) + denotes the protonated molecular ion); POS, positive ionization mode; NEG, negative ionization mode.

To decipher the physiological implications, we performed KEGG pathway enrichment analysis on the DMs using the MBROLE 2.0 web server. This analysis uncovered significant perturbations in multiple metabolic pathways (Figure 8C). A scatter plot of pathway impact further highlighted the most affected pathways (Figure 8C), which included starch and sucrose metabolism, galactose metabolism, the phosphotransferase system (PTS), pyruvate metabolism, ABC transporters, fructose and mannose metabolism, fatty acid biosynthesis, phenylalanine metabolism, lysosome function, cysteine and methionine metabolism, GABAergic synapse, and amino sugar and nucleotide sugar metabolism.

Heatmap visualization illustrated substantial remodeling across major metabolite classes (Figure 8D). Specifically, intracellular levels of organic oxygen compounds—such as D-Mannose, Sucrose, Galactinol, and Trehalose—were markedly depleted in the 1/2 MIC of ICD group compared to the control. Similarly, key organic acids and derivatives, including Phosphocreatine and Succinate, were significantly reduced. Conversely, the content of several lipids and lipid-like molecules, such as Oleic acid, Caprylic acid, Valeric acid, and Heptadecanoic acid, was notably elevated. Among benzenoids, Benzoic acid accumulated significantly, whereas S-Methyl-5′-thioadenosine levels decreased. These metabolomic perturbations indicate that *M. bovis* undergoes extensive metabolic reprogramming under ICD stress. The overall pattern—characterized by energy depletion and the accumulation of lipid and benzenoid precursors—suggests a state of metabolic dysregulation that is further explored in the discussion. These widespread metabolic disruptions, especially in energy and lipid metabolism, coincide with the transcriptional upregulation of efflux pumps and stress responses. This suggests a bacterium experiencing significant energetic and membrane stress.

## Discussion

This study presents a multifaceted investigation into the antibacterial activity and putative mechanism of action of ICD, a natural alkaloid derived from *DLF*, against *M. bovis*. Through the integration of phenotypic assays, electron microscopy, transcriptomics, and metabolomics, we have constructed a comprehensive model elucidating how ICD exerts its effects and how *M. bovis* orchestrates an adaptive response.

This study demonstrates that ICD from *DLF* exhibits bacteriostatic activity against *M. bovis*, with a MIC of 400 μg/mL and an IC_50_ of 198.04 μg/mL. In contrast, previous research reported that alkaloids from *DLF* have a MIC of 2.5 mg/mL and an IC_50_ of 1.389 mg/mL against *Klebsiella pneumoniae* (Wang et al., 2018), indicating a more potent antibacterial effect of ICD against *M. bovis*. Alkaloids from *DLF* showed MIC values of 0.35 mg/mL and 0.22 mg/mL against *Staphylococcus aureus* and *Escherichia coli*, respectively, with IC_50_ values of 0.214 mg/mL for both (Wang et al., 2017; Zhao et al., 2008). Compared to MIC of ICD against *M. bovis*, the MIC values for *S. aureus* and *E. coli* are lower, while the IC_50_ values are higher. These variations may result from differences in bacterial cell wall structures. *M. bovis*, a gram-positive, acid-fast bacillus, has a cell wall that contains peptidoglycans and unique glycolipids (Kanipe and Palmer, 2020), which may influence its susceptibility to ICD. These results highlight the potential of ICD as a phytochemical agent against mycobacteria, although further comparative studies are needed to assess its efficacy. Although the MIC of ICD exceeds the clinical thresholds of first line tuberculosis drugs like rifampicin (Nahid et al., 2016), its IC_50_/MIC ratio indicates potent, concentration-dependent antibacterial activity, a pharmacological trait often associated with bactericidal agents (Silva et al., 2016). The high MIC observed may be attributed to the limited permeability of the mycobacterial lipid-rich cell wall, a recognized barrier to plant derived compounds (Drusano, 2004). Enhancing ICD’s penetration through the mycobacterial cell wall could thus improve its therapeutic efficacy.

The phase-dependent susceptibility of *M. bovis* to ICD, with peak sensitivity during early to mid-logarithmic growth, suggests that ICD targets biosynthetic pathways active during rapid bacterial proliferation. This growth phase specificity is reminiscent of cell wall inhibitors like pyrazinamide, which exhibit enhanced efficacy against metabolically active mycobacteria (Zhang et al., 2003). Similarly, pyrolin inhibits cell growth and division during the logarithmic phase in both *Staphylococcus aureus* and *Pseudomonas aeruginosa* (Yu et al., 2008). These findings emphasize the importance of bacterial growth phases dynamics in the use of antimicrobial agents. Targeted inhibition during specific growth stages may enhance ICD’s efficacy, meriting further exploration of its therapeutic potential.

Furthermore, we observed a dose-dependent increase in OD260 nm in *M. bovis* suspensions treated with ICD. The limited nucleic acid leakage indicates partial membrane poration without complete bacteriolysis, akin to the transient pore formation by cationic antimicrobial peptides (Wimley, 2010). Additionally, ICD induced concentration-dependent changes in extracellular pH in *M. bovis* suspensions. At 1/2 MIC, the pH decrease likely reflects sustained bacterial metabolic activity, characterized by acid byproducts excretion, typical of viable mycobacteria maintaining a proton motive force for nutrient uptake (Berney and Berney-Meyer, 2017). In contrast, the pH increase at MIC and 3/2 MIC suggests membrane integrity loss, leading to leakage of alkaline cytoplasmic components. This pH shift pattern parallels the effects of ethambutol on mycobacterial membrane potential (Korycka-Machała et al., 2005), indicating potential shared targets within cell envelope maintenance systems. This parallel suggests that ICD may act through mechanisms similar to those of established antimycobacterial agents, thereby providing a foundation for further exploration into its efficacy and the biochemical pathways involved in its action.

The concentration dependent increase in electrical conductivity in *M. bovis* suspensions treated with ICD indicates progressive membrane destabilization. Elevated conductivity indicates leakage of intracellular ions, such as potassium and sodium, typically concentrated within the cytoplasm (Clementi et al., 2014). This ion leakage suggests that ICD may disrupt membrane potential or impair membrane-associated proteins, leading to a loss of cellular compartmentalization. Similar effects have been reported for various antimicrobial agents targeting bacterial membrane integrity (Grabowicz, 2018). At sub-concentration, the moderate conductivity increase suggests partial membrane poration, facilitating selective ion leakage, consistent with the action of cationic antimicrobial peptides that form transient membrane pores (Wimley, 2010). Conversely, the sharp increase in conductivity at 3/2 MIC suggests complete membrane rupture and cytoplasmic content release akin to the lytic effects of polymyxins on Gram-negative bacteria (Velkov et al., 2013). Similarly, tea tree oil has been shown to significantly elevate 260 nm absorbance and electrical conductivity in fungal hyphae (Shao et al., 2013). In *G. citri-aurantii*, membrane permeability increased with higher concentrations of citral, octanal and *α*-terpineol as indicated by constituent release, extracellular conductivity, and pH changes (Zhou et al., 2014). This ion flux likely disrupts the proton motive force, impairing energy metabolism and nutrient transport, paralleling the action of bedaquiline in tuberculosis treatment (Hards et al., 2018).

Lipids serve as essential components of the mycobacterial cell wall, contributing significantly to its impermeability and resistance against environmental stressors, including antibiotics (Topf et al., 2012). The concentration-dependent reduction in total lipid content of *M. bovis* cells exposed to ICD from *DLF* indicates that ICD disrupts cell wall-associated lipids. By depleting lipid reserves, ICD has the potential to compromise the structural integrity of the cell wall, increasing the susceptibility of *M. bovis* to host immune responses and antimicrobial agents. This mechanism aligns with the therapeutic potential of various plant-derived compounds against mycobacterial infections (Gautam et al., 2023). Furthermore, this phenomenon is consistent with the mechanisms of frontline antimycobacterial agents, such as isoniazid, which inhibit mycolic acid synthesis by targeting InhA (enoyl-ACP reductase) in the fatty acid elongation system (Schaeffer et al., 2001). The lipid loss pattern is like ethionamide’s impact on phthiocerol dimycocerosate (PDIM) biosynthesis (Kruh et al., 2008). Further investigation is needed to identify ICD’s precise molecular targets in *M. bovis* lipid metabolism. Potential pathways include fatty acid synthase (FAS) inhibition or disruption of lipid droplet formation, both involved in mycobacterial lipid regulation (Elamin et al., 2011). Additionally, exploring the synergistic effects of ICD in conjunction with existing antimycobacterial drugs could offer insights into its potential as an adjunctive therapy.

Scanning electron microscopy (SEM) and transmission electron microscopy (TEM) provide strong evidence that ICD from *DLF* exerts bactericidal activity against *M. bovis*. SEM revealed marked morphological alterations, including cell shrinkage, membrane and wall distortion or rupture, irregular overall architecture, heterogeneous cell shape, and protoplast extrusion, all of which indicate loss of cellular integrity. TEM corroborated these findings by showing plasmalemma shrinkage, rupture or absence, and dissolution of intracellular contents. Together, these ultrastructural changes indicate that ICD disrupts the cellular envelope and internal organization of *M. bovis*, leading to irreversible cell death—a defining feature of bactericidal action. These effects are consistent with mechanisms of other antimicrobials that target bacterial cell walls and membranes; for example, *β*-lactams elicit comparable outcomes such as cell lysis and protoplast release (Silhavy et al., 2010). The intracellular dissolution seen in TEM images resembles the effects of membrane-targeting agents such as polymyxins, which disrupt membrane integrity and cause cytoplasmic leakage (Velkov et al., 2013). Our results also align with prior reports showing that ICD impairs membrane energetics and nucleic acid synthesis (Wei et al., 2024), providing a coherent mechanistic explanation for its bactericidal activity. The reduction in total lipid content together with the severe ultrastructural damage observed by SEM and TEM—including cell wall distortion, membrane rupture, and loss of intracellular material—identify the mycobacterial cell envelope as a principal target. This damage likely increases permeability, perturbs energy metabolism, and culminates in irreversible cell death.

Our transcriptomic analysis provides the first comprehensive overview of the dysregulated stress response of *M. bovis* to sub-inhibitory concentrations of ICD. The identification of 66 differentially expressed genes, with a significant number being up-regulated, points to a desperate, and ultimately insufficient, countermeasure employed by the bacillus to lethal stress induced by this alkaloid. The up-regulation of genes encoding efflux pump systems and their associated transcriptional regulators emerges as a central theme in this response, representing an energetically costly effort to expel the compound which, in the context of the overall bactericidal outcome, proves inadequate. The present study identified the significant upregulation of genes associated with drug efflux, particularly those belonging to the ATP-binding cassette (ABC) transporter superfamily (*M.b_GM001808*, *M.b_GM001809*) and the MMPL (Mycobacterial Membrane Protein Large) family (*M.b_GM001674*) in *M. bovis* under ICD stress. Efflux pumps play a critical role in bacterial defense, extruding a wide range of toxic compounds, including antibiotics, from the cell (Lorusso et al., 2022). ABC transporters, which utilize ATP hydrolysis to power substrate translocation, are well-documented contributors to drug resistance in mycobacteria. For instance, the phosphate-specific transporter Pst of *M. tuberculosis* has been shown to export ciprofloxacin (Banerjee et al., 2000), and the ABC efflux pump encoded by the *Rv2686c-Rv2688c* operon confers resistance to fluoroquinolones in *Mycobacterium smegmatis* (Pasca et al., 2004). More recently, the ABC transporter *Rv1273c* was characterized as a multidrug efflux pump with broad substrate specificity (Cox et al., 2014). The coordinated upregulation of ABC transporter genes in *M. bovis* under ICD stress strongly implies their direct involvement in attempting to expel the compound, thereby reducing its intracellular concentration and mitigating its toxic effects.

The up-regulation of efflux pump genes was accompanied by increased expression of several TetR family transcriptional regulators (*M.b_GM003352*, *M.b_GM001807*, *M.b_GM003253*). TetR regulators commonly control efflux pump expression in response to environmental stressors, including antibiotics (Patil et al., 2025). These regulators typically function as repressors that are inactivated upon binding a specific ligand, leading to derepression of downstream target genes, often encoding efflux systems. For instance, in *Mycobacterium abscessus*, a TetR regulator (*MAB_2299c*) controls adjacent efflux pump genes, and its mutation confers cross-resistance to clofazimine and bedaquiline (Richard et al., 2019). Similarly, the *M. tuberculosis* TetR regulator *Rv1255c* enhances isoniazid tolerance under hypoxia (Rouquette-Loughlin et al., 2002). The concurrent up-regulation of TetR genes and efflux pump operons, such as the *M.b_GM001807-1808-1809* cluster, suggests the existence of a regulatory circuit in which ICD might act as an inducer, potentially binding to a TetR repressor and thereby triggering the expression of adjacent ABC transporter genes. Further investigation is warranted to elucidate the precise regulatory mechanism.

Furthermore, the up-regulation of the Small Multidrug Resistance (SMR) protein gene *M.b_GM003252* and its associated DoxX (*M.b_GM003251*) gene reinforces the efflux hypothesis. SMR proteins are known to confer resistance to lipophilic cations through proton-dependent efflux (Bay and Turner, 2009; Jack et al., 2000). The significant enrichment of KEGG pathways related to ABC transporters and two-component systems further supports the notion that membrane transport and adaptive signal transduction are critical to the ICD stress response. Conversely, the down-regulation of the ESX secretion system-associated virulence factor gene *M.b_GM003826* (EspC) is noteworthy. The ESX system is essential for mycobacterial pathogenesis and intracellular survival (Gcebe et al., 2016; MacGurn and Cox, 2007). This down-regulation may represent a trade-off, where the bacterium reallocates energy resources from virulence factor production toward survival mechanisms like efflux and detoxification under direct chemical stress (Meylan et al., 2017). A similar repression of virulence genes under antibiotic pressure has been observed in other pathogens (Alam et al., 2016; Fisher et al., 2017), indicating a potential shift to a persistence-oriented state.

The GO and KEGG analyses revealed a broad impact on metabolic processes, including the biosynthesis of secondary metabolites, amino acids, and fatty acids, as well as pathways for the degradation of various carbon sources. This widespread metabolic rewiring likely reflects both the direct inhibitory effects of ICD on certain cellular targets and the compensatory adjustments required to fuel the energy-demanding efflux processes and maintain redox homeostasis (Seyedsayamdost, 2019; Rosas Olvera et al., 2017). Our transcriptomic data strongly suggest that *M. bovis* employs a multi-faceted defense against ICD, primarily centered on the up-regulation of drug efflux systems (ABC transporters, MMPL, SMR) governed by TetR family transcriptional regulators. The concomitant shift in metabolic pathways indicates a significant energetic burden associated with this adaptive response. These findings not only shed light on the potential mechanism of action of and resistance to ICD but also identify *M.b_GM001807* (TetR) and the *M.b_GM001808-1809* (ABC transporter) operon as high-priority candidates for future genetic studies, such as gene knockout and complementation experiments, to definitively confirm their role in ICD tolerance.

The profound physical damage to the cell envelope, as evidenced by electron microscopy and physiological assays, was reflected in a state of catastrophic metabolic dysregulation. The comprehensive metabolomic analysis of *M. bovis* under sub-inhibitory stress from the natural alkaloid ICD revealed profound metabolic alterations. LC–MS analysis identified 1,160 and 672 differential metabolites in positive and negative ion modes, respectively, underscoring a widespread state of metabolic dysregulation under ICD stress. Pathway enrichment analysis indicated critical disruptions in energy homeostasis and membrane integrity, which collectively point to a failure of metabolic homeostasis rather than a successful compensatory survival strategy. These included alterations in starch and sucrose metabolism, galactose metabolism, the phosphotransferase system, ABC transporters, and fatty acid biosynthesis. A central finding was the significant perturbation of carbon and energy metabolism. The marked decrease in intracellular sugars like D-Mannose, Sucrose, Galactinol, and Trehalose suggested a rapid mobilization of carbon sources to fuel emergency responses. Trehalose, a critical disaccharide in mycobacteria, serves as a carbon reserve and a key component of the cell wall glycolipids, as well as a stress protectant. Its depletion could indicate consumption to generate energy or reinforce the cell wall structure in the face of ICD-induced damage (Vanaporn and Titball, 2020). Concurrently, the decrease in phosphocreatine, a high-energy phosphate buffer, and succinate, a central TCA cycle intermediate, signaled a high energy demand and potential disruption of oxidative phosphorylation (Liu et al., 2021). The observed enrichment of the pyruvate metabolism and phosphotransferase system (PTS) pathways suggests a reprogramming of sugar uptake and central carbon metabolism to optimize ATP production during the energy crisis.

The metabolomic perturbations identified in this study highlight significant metabolic dysregulation in *M. bovis* under ICD stress, marked by a critical breakdown in anabolic repair processes. A central, paradoxical finding was the simultaneous accumulation of fatty acids (e.g., oleic, caprylic, valeric acid) and specific sugars (e.g., sucrose, trehalose), alongside a decrease in total cellular lipid content and cell wall damage. We interpret this not as a successful adaptive response but as evidence of a biosynthetic blockade. The buildup of oleic acid—a known precursor for mycolic acid synthesis (Glickman et al., 2000)—strongly indicates a disruption in the downstream machinery needed to incorporate these precursors into the complex cell wall structure. Accumulation of precursors signaling pathway disruption is a recognized hallmark of metabolic stress in microbes (Sharma and Jha, 2021). This results in a futile metabolic cycle: the bacillus mobilizes or catabolizes resources to generate precursors, but ICD-induced physical damage and energy deficits prevent their efficient use for structural repair. Such futile cycling, which depletes cellular reserves without achieving homeostasis, is a known driver of bactericidal outcomes under antibiotic stress (Lobritz et al., 2015).

The metabolomic profile reveals a pronounced catabolic response overshadowed by a terminal anabolic blockade. The accumulation of fatty acids alongside the depletion of key energy metabolites, such as phosphocreatine and succinate, suggests a shift toward lipid utilization to meet energy demands (Muñoz-Elías and McKinney, 2005). However, the simultaneous presence of these catabolic markers with the buildup of biosynthetic precursors, like oleic acid for mycolic acids, and a reduction in total lipids signifies a critical failure in anabolism. The bacillus struggles to efficiently convert these mobilized resources into cell wall repair, resulting in a futile cycle that depletes energy and precursors without restoring structural integrity (Lobritz et al., 2015). This interpretation aligns with the observed ultrastructural damage and supports a model where ICD functions as a disruptive, multi-target bactericidal agent.

Highlighting the antibacterial mechanism of ICD within the framework of established therapeutics emphasizes its uniqueness. A comparative analysis of the transcriptional and metabolic profiles of *M. tuberculosis* complex pathogens exposed to rifampicin reveals fundamentally distinct modes of action (Boshoff et al., 2004; Gumbi et al., 2019). Rifampicin directly inhibits RNA polymerase, leading to a global transcriptional shutdown characterized by a strong induction of the stringent response and a significant downregulation of ribosomal protein and tRNA synthesis genes (Betts et al., 2003; Boshoff et al., 2004). In contrast, ICD treatment neither triggers a significant stringent response nor suppresses ribosomal gene expression. Instead, ICD stress is marked by a profound dysregulation of cell envelope biosynthesis and lipid homeostasis. Metabolically, while rifampicin exposure causes rapid depletion of nucleotide pools due to halted RNA synthesis (Jain et al., 2022), our data for ICD reveal no such pattern, instead indicating energetic collapse and dysfunctional precursor accumulation. This distinct profile from a transcription-targeting antibiotic strongly supports our model that ICD primarily disrupts the bacterial cell envelope through a multi-targeted assault, representing a mechanism distinct from that of first-line drugs.

Comparing ICD’s mechanism to rifampicin at phenotypic and molecular levels reveals significant differences. Phenotypically, ICD induces rapid, concentration-dependent membrane damage, characterized by immediate ion leakage and release of cellular constituents, leading to a bactericidal effect. This sharply contrasts with rifampicin’s primarily bacteriostatic effect, where a slow decline in viability is well-documented in kill-kinetics studies (Gumbo et al., 2007). Molecularly, rifampicin targets the RNA polymerase beta subunit, with structural studies precisely mapping its binding site and transcription inhibition mode (Campbell et al., 2001). In contrast, our study does not pinpoint a single protein target for ICD. However, multi-omics data consistently indicate the mycobacterial cell envelope as the focal point of ICD’s activity. We propose a mechanism involving lipid depletion, membrane potential dissipation, and disrupted cell wall precursor metabolism. This shares functional similarities with drugs like bedaquiline, which disrupts bacterial energetics through ionophoric effects (Hards et al., 2018), and ethambutol, known to affect membrane integrity alongside its cell wall target (Korycka-Machała et al., 2021). Nonetheless, the distinct transcriptional and metabolic signatures we discovered suggest that ICD operates through a novel pathway within this broad target space, setting it apart from these established agents.

The observed metabolic changes and alterations in membrane lipids, such as 1-palmitoyl-2-oleoyl-phosphatidylglycerol (POPG), are interpreted here not as coordinated adaptations, but as results of dysfunctional homeostasis and failed repair attempts. Although bacteria can remodel their cell envelopes in response to antimicrobial stress (Zhuang et al., 2022; Liu et al., 2021), the severe damage and energy depletion caused by ICD suggest these changes are inefficient and disordered. The accumulation of fatty acid precursors makes them available for remodeling; however, physical damage to the biosynthetic machinery likely prevents the formation of a functionally coherent membrane. Consequently, any remodeling that occurs is unlikely to effectively strengthen the cell envelope or reduce ICD permeability. This interpretation of an overwhelmed cellular state aligns with the concurrent enrichment of the ABC transporters pathway. The upregulation of efflux pumps, instead of indicating a successful tolerance mechanism, more likely represents a desperate and energetically costly countermeasure that, in the face of overwhelming damage, ultimately fails to prevent the intracellular accumulation of ICD and the ensuing bactericidal outcome. Additional evidence of systemic failure is seen in the modulation of methylation cycle metabolites. The accumulation of S-Adenosyl-L-homocysteine (SAH) alongside the reduction of S-Methyl-5′-thioadenosine suggests a significant disruption in the methionine cycle (Ogawa et al., 2016). This disturbance likely indicates a breakdown in essential cellular signaling and methylation processes, which are crucial for the regulation of metabolism and virulence (Bibb and Hatfull, 2005), rather than a “sophisticated regulatory response.” Such dysregulation would severely impair the cell’s capacity to initiate a coherent transcriptional and translational response to stress.

The antibacterial mechanism of ICD described here was determined in an axenic culture system. This approach delineates ICD’s direct bactericidal effects and the primary metabolic disruptions it causes in *M. bovis*, but it does not capture host-mediated modulation. Future studies using ex vivo macrophage infection models are needed to validate these findings and to determine whether ICD can penetrate and act on intracellular bacilli. Such models would also clarify ICD’s effect on efflux pump upregulation within phagosomes and its potential to synergize with host-derived stresses (Zimmermann et al., 2021). Finally, *in vivo* efficacy studies are required to assess ICD’s therapeutic potential, particularly when delivered with advanced formulations designed to overcome its current pharmacokinetic limitations (Pinheiro et al., 2023). In summary, our multi-omics data support a unified model of ICD’s bactericidal action. ICD inflicts irreparable physical damage to the cell envelope, which in turn triggers a catastrophic metabolic crisis. The observed transcriptional and metabolomic responses—including efflux pump expression, lipid accumulation, and signaling pathway disruption—constitute the bacillus’s failed attempt to restore homeostasis under overwhelming assault. These responses are rendered futile by the combined effects of energy depletion and disruption of key biosynthetic and regulatory networks. Thus, we conclude that ICD kills *M. bovis* by initiating a self-amplifying cycle of damage and dysregulation that exceeds the bacterium’s compensatory capacity. This multi-targeted mechanism, distinct from conventional antibiotics, underscores ICD’s potential as a promising lead compound for overcoming drug resistance.

The phase-dependent antibacterial activity and the multi-target mechanism of action of ICD, as elucidated in this study, provide a rational basis for its potential integration into therapeutic strategies. Our findings indicate that ICD exerts its most potent activity against metabolically active *M. bovis*, a trait it shares with certain frontline tuberculosis drugs (Zhang and Mitchison, 2003). This specific activity profile suggests that ICD could be strategically deployed during the initial, intensive phase of a combination therapy regimen, with the goal of rapidly diminishing the population of metabolically active bacilli. To address the challenge of its relatively high MIC, potentially a consequence of the formidable mycobacterial cell wall barrier (Jankute et al., 2015), the development of advanced drug delivery systems—such as liposomal or nanoparticle-based formulations—could be pursued to enhance targeted delivery and intracellular accumulation (Pinheiro et al., 2023). Furthermore, the concomitant upregulation of efflux pumps, while ultimately insufficient to prevent death in our model, underscores a tangible risk of resistance emergence during prolonged sub-lethal exposure, reinforcing the imperative for its use in combination with other antibacterial agents. While these translational propositions necessitate future validation, they contextualize our mechanistic insights within a practical framework, positioning ICD as a promising candidate for future anti-mycobacterial drug development.

Our study indicates that ICD primarily targets the mycobacterial cell envelope, compromising membrane integrity and disrupting lipid homeostasis. This disruption leads to ion leakage, dissipation of the proton motive force, and ultimately, cell death. The multi-omics mechanistic insights presented here establish a foundational understanding of ICD’s direct antibacterial activity, providing a robust framework for its future evaluation in more complex, host-relevant systems. Although *M. bovis* mounts a multifaceted defense, including efflux pump upregulation and metabolic shifts, these responses are ultimately overwhelmed by ICD’s assault.

## Data Availability

The original contributions presented in the study are publicly available. The transcriptomic data can be found in the NCBI Sequence Read Archive (SRA) under accession number PRJNA1320159. The metabolomics data can be found in the MetaboLights repository under accession number MTBLS13441.
