# Supplementary material for: Integrated transcriptomic and metabolomic analyses reveal the antibacterial mechanism of isocorydine against Mycobacterium bovis
Source: Front Microbiol. 2025 Dec 11;16:1717499. doi: 10.3389/fmicb.2025.1717499 (PMC12740009; doi:10.3389/fmicb.2025.1717499)
Supplement: Supplementary file 1 [file Data_Sheet_1.docx]

Supplementary Material

# Detection of the isocorydine concentration

The isocorydine concentration were 99.67% pure as judged by analysis with an HPLC system (HPLC: Agilent Technologies LC 1260 Infinity II series). Chromatography column: Kromasil 100-5-C18 (4.6*250 mm, 5 μm). Mobile phase: A-Acetonitrile, B-0.2% Acetic acid in water (Adjust the pH to 5.1 with diethylamine). Chromatographic conditions: Column temperature T=35 ℃. Flow rate V=1.0 ml/min, λ=268 nm. Injection volume: 20.0 uL. Column pressure: 199 bar. Gradient elution: A,15%-25%, 20min, 25%-80%, 10 min. The retention time is (RT) 16.784 min, the %Area is 99.67 (Figure S1).

# Growth analysis with different concentration of isocorydine on 7H10

*Mycobacterium bovis* CVCC68002 pre-cultured in 7H9 medium supplemented with 10% OADC were collected, washed twice, and transferred to 7H10 medium containing different concentration of isocorydine and rifampicin. When 100, 200, 300, 400 μg/μL isocorydine or 32 μg/μL rifampicin was added in 7H10 medium, *M. bovis* growth was observed at different times. For growth assay on 7H10 solid medium, cells grown to the exponential phase in liquid 7H9 medium with 10% OADC were collected and diluted to an appropriate density (Figure S2).

# Analysis of bacteriostasis rate and IC50

To evaluate the bacteriostatic effect and determine the half-maximal inhibitory concentration (IC_50_) of ICD from *DLF* on *M. bovis*, optical density (OD) at 600 nm was measured. Measurements were taken after 4 weeks of growth without ICD (A_0_) and with ICD (An). Additionally, the OD of the medium containing ICD but without bacteria was recorded (Am) to account for ICD 's intrinsic absorbance. The bacteriostasis rate (η) was calculated as follows: η=(A_0_-A_n_-A_m_)/A_0_ ×100%. Here, A_0_ is the OD at 600 nm for the control at 4 weeks, A_n_ is the OD with ICD at 4 weeks, and A_m_ is the OD of the medium without cells. The IC_50_ was then determined using the Reed-Muench method as described by Pizzi (1950) in Table S1.

$$\boldsymbol{\eta}\boldsymbol{=}\left( \frac{\mathbf{A}\mathbf{0}\mathbf{-A}\mathbf{n}\mathbf{-A}\mathbf{m}}{\mathbf{A0}} \right)\boldsymbol{100\%}$$

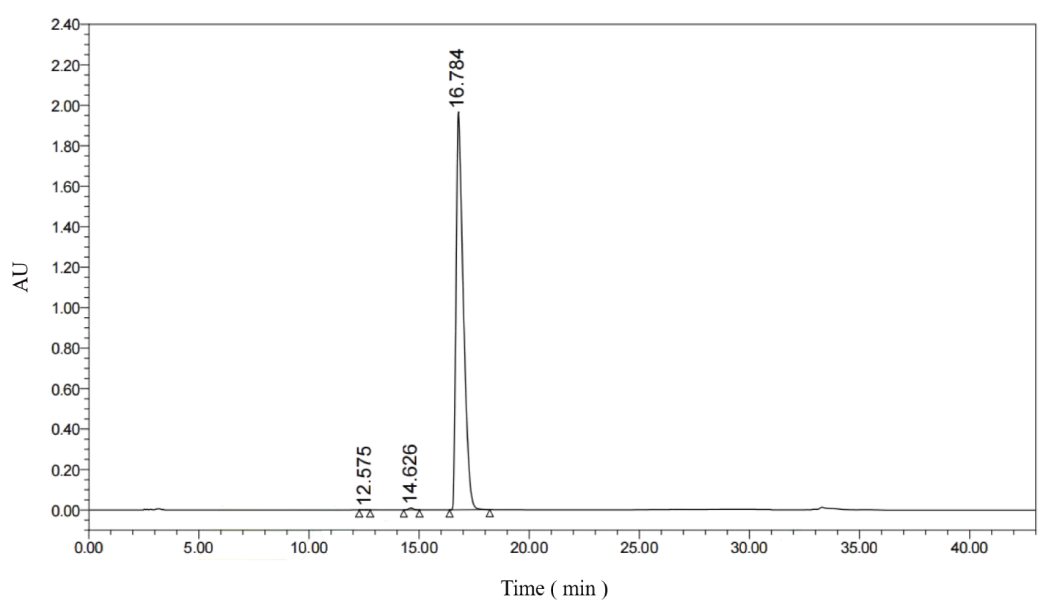


**Supplementary Figure S1.** The purity of isocorydine was detected by HPLC system
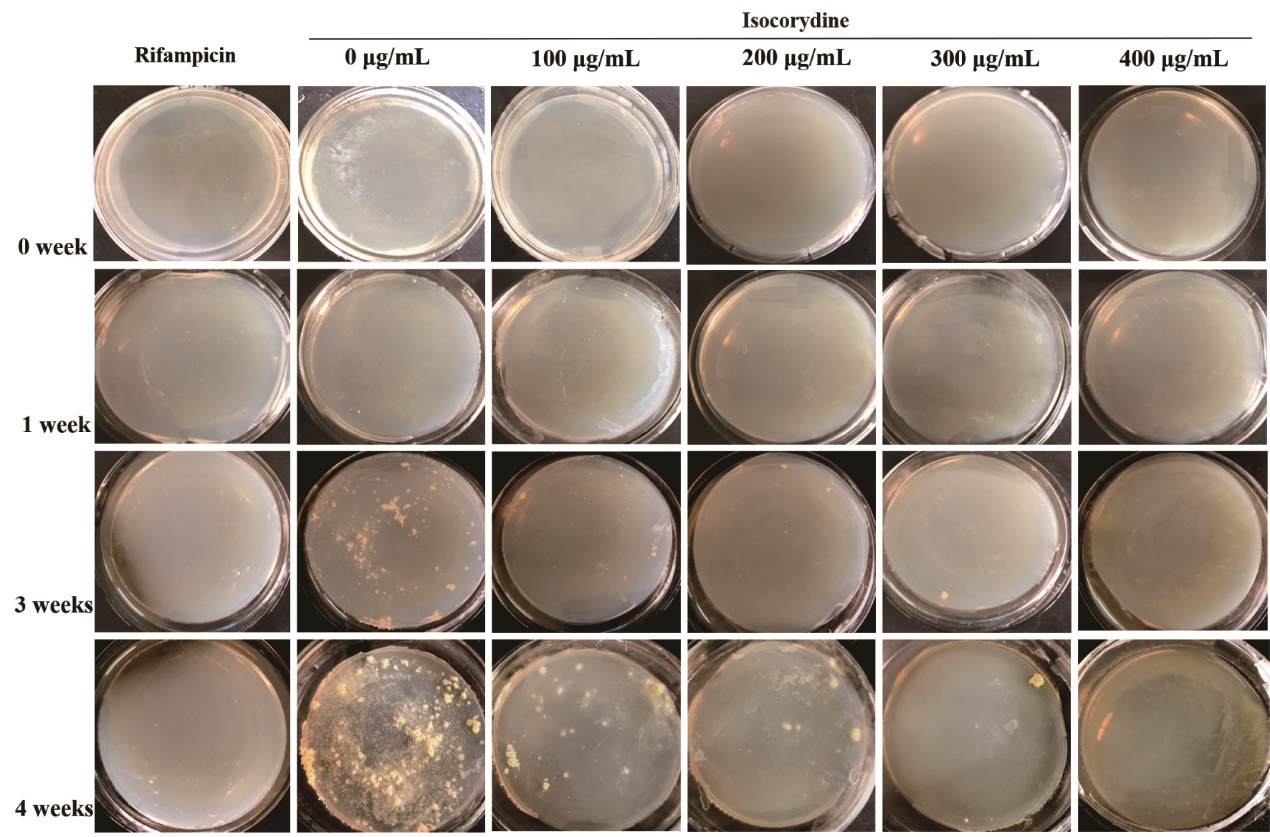


**Supplementary Figure S2.** Growth analyses of *M. bovis* CVCC68002 strains on solid plate with different concentration of isocorydine of 7H10 medium. The growth assay was performed in duplicate (with the same results) and one representative result is shown.

**Supplementary Table S1** Bacteriostasis rate and IC_50_ determination of isocorydine against *M. bovis*.

| Dose (μg·mL) | 0 | 50 | 100 | 150 | 200 | 250 | 300 | 350 | 400 |
| --- | --- | --- | --- | --- | --- | --- | --- | --- | --- |
| Bacteriostasis rate (%) | 0 | 0 | 14.03 | 26.07 | 42.67 | 51.12 | 73.69 | 87.76 | 100 |
| IC_50_ | 198.04 | | | | | | | | |

**Supplementary Table S2** Primers used in this study

| Primer | Sequence（5’to 3’) | Description |
| --- | --- | --- |
| RT-GM3251-F | CTTTCGGTTGGTATACGGGCT | Forward primer for confirming transcription of *M.b_GM003251* |
| RT-GM3251-R | GGGCCAGGATCCAAATTCGA | Reverse primer for confirming transcription of *M.b_GM003251* |
| RT-GM1809-F | CGGTATTGCGCGTCGAATTAT | Forward primer for confirming transcription of *M.b_GM001809* |
| RT-GM1809-R | TGCATCCGGTTTCCTTTCGTA | Reverse primer for confirming transcription of *M.b_GM001809* |
| RT-GM0821-F | GAGTTTGACGGCGGCTTTAC | Forward primer for confirming transcription of *M.b_GM000821* |
| RT-GM0821-R | GGTCATCATGTCGAGGTTCCA | Reverse primer for confirming transcription of *M.b_GM000821* |
| RT-GM3826-F | GCTCACAGTTCAACGACACG | Forward primer for confirming transcription of *M.b_GM003826* |
| RT-GM3826-R | CCTCGCTATATATTTTCGCCGC | Reverse primer for confirming transcription of *M.b_GM003826* |
| RT-GM3353-F | GGTTGATGCTGATGGTGGAAC | Forward primer for confirming transcription of *M.b_GM003353* |
| RT-GM3353-R | GTTGAAGTCTAAGTTGGCGGC | Reverse primer for confirming transcription of *M.b_GM003353* |
| RT-GM3352-F | AGCTGTTCGGTCACATCACC | Forward primer for confirming transcription of *M.b_GM003352* |
| RT-GM3352-R | CGTTCCATCTCCGCGACTAG | Reverse primer for confirming transcription of *M.b_GM003352* |
| RT-GM2863-F | GATGAGCCGGTAAAACGCAC | Forward primer for confirming transcription of *M.b_GM002863* |
| RT-GM2863-R | GATGGTGCTGGTCGTAGTGTC | Reverse primer for confirming transcription of *M.b_GM002863* |
| RT-GM1674-F | CGAAATCTTGCAGCGCCAATA | Forward primer for confirming transcription of *M.b_GM001674* |
| RT-GM1674-R | CGAATCGGCCTGAAGAAATCG | Reverse primer for confirming transcription of *M.b_GM001674* |
| RT-GM3252-F | ATACCTCTTGTGCGCGATCTT | Forward primer for confirming transcription of *M.b_GM003252* |
| RT-GM3252-R | GCGATGCCATAACCCACTAGA | Reverse primer for confirming transcription of *M.b_GM003252* |
| RT-GM3253-F | GCTCACCACCGTCTACCTG | Forward primer for confirming transcription of *M.b_GM003253* |
| RT-GM3253-R | CAGCGCGAGTAGACCATCTG | Reverse primer for confirming transcription of *M.b_GM003253* |
| RT-GM1808-F | TTCAGGCCGTACAGTTCATCC | Forward primer for confirming transcription of *M.b_GM001808* |
| RT-GM1808-R | CGGCATCACGTTACTGATCCA | Reverse primer for confirming transcription of *M.b_GM001808* |
| RT-GM1807-F | ATCCTTGATCTCAGGCTCCGA | Forward primer for confirming transcription of *M.b_GM001807* |
| RT-GM1807-R | CCATGACCACACCCATCAGTT | Reverse primer for confirming transcription of *M.b_GM001807* |
| RT-GM0824-F | GCCAAGGACACGCGATGT | Forward primer for confirming transcription of *M.b_GM000824* |
| RT-GM0824-R | TCTCCCGTTTCTCGGATGG | Reverse primer for confirming transcription of *M.b_GM000824* |

# References

Pizzi, M. (1950). Sampling variation of the fifty percent end-point, determined by the Reed-Muench (Behrens) method. *Hum. Biol*. 22:151–190.
